# Dual role of GRHL3 in bladder carcinogenesis depending on histological subtypes

**DOI:** 10.1002/1878-0261.13623

**Published:** 2024-03-02

**Authors:** Franziska C. Lammert, Julia Pannhausen, Erik Noetzel, Florian Friedland, Julia Wirtz, Yannick Herfs, Sophie Leypold, Lin Gan, Ralf Weiskirchen, Tician Schnitzler, Ruth Knüchel, Jochen Maurer, Danny D. Jonigk, Michael Rose, Nadine T. Gaisa

**Affiliations:** ^1^ Institute of Pathology, University Hospital RWTH Aachen University Germany; ^2^ Center for Integrated Oncology Aachen Bonn Cologne Duesseldorf (CIO ABCD) Germany; ^3^ Institute of Biological Information Processing 2 (IBI‐2), Mechanobiology, Forschungszentrum Jülich GmbH Germany; ^4^ IZKF Aachen Medical Faculty of the RWTH Aachen University Germany; ^5^ Institute of Molecular Pathobiochemistry, Experimental Gene Therapy and Clinical Chemistry (IFMPEGKC), University Hospital RWTH Aachen University Germany; ^6^ Department of Obstetrics and Gynecology University Hospital Aachen Germany; ^7^ German Center for Lung Research, DZL, BREATH Hanover Germany; ^8^ Institute of Pathology, University Hospital University of Ulm Germany

**Keywords:** bladder cancer, GRHL3, oncogene, RHO GTPase, squamous cell carcinoma, tumor suppressor gene

## Abstract

The effect of grainyhead‐like transcription factor 3 (GRHL3) on cancer development depends on the cancer subtypes as shown in tumor entities such as colorectal or oral squamous cell carcinomas. Here, we analyzed the subtype‐specific role of GRHL3 in bladder carcinogenesis, comparing common urothelial carcinoma (UC) with squamous bladder cancer (sq‐BLCA). We examined GRHL3 mRNA and protein expression in cohorts of patient samples, its prognostic role and its functional impact on tumorigeneses in different molecular and histopathological subtypes of bladder cancer. We showed for GRHL3 a reverse expression in squamous and urothelial bladder cancer subtypes. Stably GRHL3*‐*overexpressing EJ28, J82, and SCaBER *in vitro* models revealed a tumor‐suppressive function in squamous and an oncogenic role in the urothelial cancer cells affecting cell and colony growth, and migratory and invasive capacities. Transcriptomic profiling demonstrated highly subtype‐specific *GRHL3*‐regulated expression networks coined by the enrichment of genes involved in integrin‐mediated pathways. In SCaBER, loss of ras homolog family member A (RHOA) GTPase activity was demonstrated to be associated with co‐regulation of eukaryotic translation initiation factor 4E family member 3 (*EIF4E3*), a potential tumor suppressor gene. Thus, our data provide for the first time a detailed insight into the role of the transcription factor *GRHL3* in different histopathological subtypes of bladder cancer.

## Graphical abstract

In the urinary bladder, GRHL3 function in cancer development depends on the histological subtype and molecular background. In urothelial carcinomas (UC), GRHL3 is strongly expressed, whereas it is downregulated in squamous cell carcinomas (SCC). *In vitro* opposite functions were confirmed in GRHL3‐overexpressing models for SCC and UC affecting cell growth, migration, invasion, focal adhesion, and GTPase activity in a subtype‐specific manner.

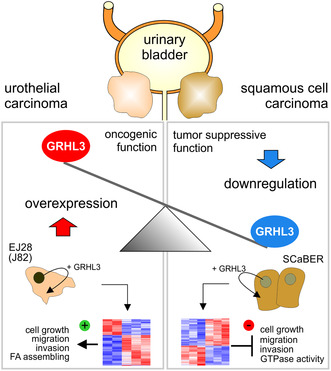

**Abbreviations**
ARHGEF19: rho guanine nucleotide exchange factor 19
BASQ: basal/squamous‐like
BLCA: bladder cancer
BP: biological processes
CB: cytoskeleton buffer
CC: cellular compartments
DEG: differentially expressed gene
EIF4E3: eukaryotic translation initiation factor 4E family member 3
EMT: epithelial‐to‐mesenchymal transition
FA: focal adhesion
FC: fold change
GO: gene ontology
GRHL: Grainyhead‐like transcription factor
IRS: immunoreactive score
MF: molecular functions
MIBC: muscle‐invasive bladder cancer
MIX: urothelial carcinoma with squamous differentiation
NAC: neoadjuvant chemotherapy
NMIBC: non‐muscle‐invasive bladder cancer
NU: normal urothelium
OS: overall survival
RFS: relapse‐free survival
RHOA: ras homolog family member A
RND3: rho family GTPase 3
RT: room temperature
SCC: pure squamous cell carcinoma
sq‐BLCA: pure squamous cell carcinomas and urothelial carcinoma with squamous differentiation
sq‐Cis: squamous cell carcinoma *in situ*
sq‐Meta: squamous metaplasia
TCGA: The Cancer Genome Atlas
TMA: tissue microarrays
UC: urothelial carcinoma

## 1 Introduction

Grainyhead‐like transcription factor 3 (GRHL3) belongs to the *GRHL* transcription factor family, which also includes GRHL1 and GRHL2 [1]. *GRHL* genes were first detected in a Drosophila mutant, which showed incomplete neural tube closure, epithelial barrier disorders, and a granular structure of the head caused by *GRHL* deficiency and thus became the eponym of Grainyhead gene [2]. Beyond that, GRHL3 modulates wound healing [1] and affects the terminal differentiation of urothelial cells [3]. In the urothelium of the bladder, GRHL3 is strongly expressed, especially in umbrella cells proposing an important function for maturation of umbrella cells [3]. This was supported by identifying target genes of GRHL3 expression in the healthy bladder, such as cell adhesion molecules [4] and uroplakin II, known to be crucial for the urinary bladder barrier but downregulated in *GRHL3*‐deficient mice [3, 5].

In carcinogenesis, the GRHL transcription factor family has been reported to exert both tumor‐suppressive and oncogenic functions, dependent on the cancer type and the tissue of origin [6]. In squamous cell carcinomas (SCC) of the skin, in oral SCC, head and neck SCC as well as breast cancer, GRHL3 has been demonstrated to mediate tumor‐suppressive effects [6, 7, 8, 9, 10]. The tumor‐suppressive function of GRHL3 in the skin has been linked with the transcriptional regulation of the direct GRHL3 target gene *PTEN* to control the PI3K/mTOR pathway [11]. GRHL2 is known to play a protumorigenic role, for example, in oral SCC [12]. However, GRHL3 can also function as tumor promoter, particularly in colorectal carcinoma [13] and diffuse large B‐cell lymphomas [14].

In bladder cancer, the role of GRHL3 is less well known, especially in the context of molecular and histological subtypes. Like most carcinomas, urinary bladder cancer is a heterogeneous disease comprising the most frequent urothelial cancers but also rare subtypes like squamous‐differentiated bladder cancers [15] known to show different biological and clinical aspects [16, 17]. SCC occurs in approximately 3% of all bladder cancers, with a squamous differentiation (MIX) in 15%. Pure SCC is associated with a more aggressive phenotype compared with urothelial carcinoma (UC) [18, 19, 20, 21]. Recently, Wezel and colleagues studied *GRHL3* in urothelial cancer development showing an impaired invasion and migration potential of *GRHL3* overexpressing urothelial cancer cells [22]. Apart from that, no further studies of *GRHL3* in bladder cancer have been described so far, and the role of *GRHL3* in sq‐BLCA remains unknown. Since opposing functions of GRHL3 depending on cancer type and/or differentiation status have been demonstrated in previous studies of other cancer entities [6, 8], we aimed to characterize for the first time GRHL3 expression, function and associated molecular downstream targets and pathways in urothelial versus squamous‐differentiated bladder cancer.

## 2 Materials and methods

### 2.1 Patient samples

Overall, our retrospective study cohort comprised *n* = 687 patient samples (archive of the Institute of Pathology and the RWTH centralized Biomaterial Bank (RWTH cBMB) or collected by the German Study Group of Bladder Cancer (DFBK e.V.)) diagnosed with squamous metaplasia (sq‐Meta; *n* = 107), squamous carcinoma *in situ* (sq‐Cis; *n* = 9), pure squamous cell carcinoma (SCC; *n* = 160), urothelial carcinoma (UC; *n* = 103), and urothelial carcinoma with squamous differentiation (MIX; *n* = 98). Normal urothelium of cystectomies (NU; *n* = 103) served as reference tissue. For comparing non‐muscle‐invasive bladder cancer (NMIBC) and its possible progression to muscle‐invasive bladder cancer (MIBC), a previously published, meticulously histologically characterized cohort of NMIBC (*n* = 107) with patients treated at the RWTH Aachen University and the LMU Munich University Hospitals between 2012 and 2018 was also included [23, 24]. Experiments were in accordance with the regulations of the centralized Biomaterial Bank (RWTH cBMB) and the Institutional Review Board (IRB)‐approved study and protocols of the Medical Faculty of RWTH Aachen University (EK 286/11, EK 206/09) as well as the Declaration of Helsinki. The experiments were undertaken with the understanding and written consent of each subject. Patients with sq‐Meta within the urethra or trigonum were excluded since metaplasia can occur physiologically and inflammation‐independent in these parts of the bladder [25]. Tissue microarrays (TMA) of patient samples (*n* = 396) were constructed with TMArrayer (Pathology Devices, Westminster, MD, USA) as described previously [26]. The characteristics of patient samples are summarized in Table S1 and for the NMIBC cohort in Table S2.

### 2.2 TCGA BLCA data set

Public BLCA data sets from the Cancer Genome Atlas (TCGA) [27] network including RNASeqV2 data (level 3) of tumor and normal tissue samples were sub‐classified as described by Robertson et al. [28] and Kamoun et al. [17]. RNASeqV2 data from BLCA tumor samples can be explored using the cBio Cancer Genomics Portal (http://cbioportal.org) [29, 30].

### 2.3 Cell lines and reagents

For *in vitro* studies, human bladder cell lines EJ28 (urothelial cell carcinoma; RRID:CVCL_5983), J82 (urothelial cell carcinoma; RRID:CVCL_0359), and SCaBER (squamous cell carcinoma; RRID:CVCL_3599) were used. J82 were originally obtained from the American Type Culture Collection (ATCC, Manassas, VA, USA). EJ28 were a gift from Dr Alexander Buchner (LMU München, Germany), and SCaBER was kindly provided by Prof Wolfgang Schulz (Düsseldorf University Hospital, Germany). EJ28 cells were cultured with RPMI 1640 medium, J82 and SCaBER cells in DMEM high glucose medium (Thermo Fisher Scientific, Waltham, MA, USA), both supplemented with 10% fetal bovine serum (Capricorn Scientific, Ebsdorfergrund, Germany) and LGPS (200 mm l‐glutamine, 50 U·mL^−1^ penicillin, 50 mg·L^−1^ streptomycin; Thermo Fisher Scientific), at 37 °C in 5% CO_2_. Test for potential mycoplasma infections was done on a regular basis. All cell lines have been authenticated based on single‐nucleotide polymorphism (SNP) typing within the last 3 years and were already available at the Institute of Pathology RWTH Aachen University.

### 2.4 Nucleic acid extraction

Prior to RNA extraction, tissue was manually micro‐dissected. RNA isolation of FFPE bladder tissues (*n* = 82) was performed using the Maxwell® 16 LEV RNA FFPE kit (Promega, Mannheim, Germany) and of cryo bladder tissues (*n* = 102) using Maxwell® 16 LEV simplyRNA Tissue Kit (Promega) according to the manufacture's protocols (ANP). cDNA synthesis was accomplished with the reverse transcription system (Promega Kit A3500), using 1 μg of total RNA.

### 2.5 Quantitative real‐time reverse transcription PCR

mRNA expression of *GRHL3* was quantified by real‐time qPCR (RT‐qPCR) on Thermal‐Cycler C1000 Touch (Bio‐Rad, Munich, Germany), using the iQ™ SYBR® Green Supermix (Bio‐Rad). Samples were measured in triplicate. Relative expression was quantified using the help of $2^{-\Delta \Delta C_{t}}$ method with *GAPDH* serving as reference gene. Primer sequences and PCR conditions are described in Table S3.

### 2.6 GRHL3 immunohistochemistry

Immunohistochemical staining of GRHL3 protein was performed with Dako EnVision FLEX system (K8000; Agilent Dako, Waldbronn, Germany) as previously described [31] with slight modifications: FFPE sections (4 μm) were dried overnight (37 °C). Dewaxing and heat‐induced antigen retrieval were performed in 10 mm citrate buffer (pH 6.0), using Dako PT Link, at 95 °C for 20 min. Endogenous peroxidase was blocked by incubation with EnVision FLEX peroxidase blocking reagent. The tissue sections were incubated at room temperature for 1 h with a primary rabbit monoclonal anti‐human GRHL3 antibody (1 : 1000, ab221058; Abcam, Cambridge, UK). The primary antibody binds a polymer strand including secondary antibody and a horseradish peroxidase (HRP) enzyme. Adding the HRP substrate diaminobenzidine (DAB) chromogen solution visualizes the binding of the primary antibody via oxidation of DAB.

GRHL3 protein expression was analyzed by applying the semi‐quantitative immunoreactive score (IRS) according to Remmele and Stegner [32].

### 2.7 Immunoblotting

Immunoblot analyses were performed according to previous publications [33, 34] and modified as follows: Incubation with primary monoclonal anti‐GRHL3 (1 : 200, TA810684; OriGene Technologies, Rockville, MD, USA) or anti‐Rho (1 : 1000, #8789, Cell Signaling Technology, Danvers, MA, USA) was performed for 2 h at room temperature and with primary monoclonal anti‐GAPDH (1 : 4000, 14C10; Cell Signaling Technology) for 1 h at 4 °C. For raw data of western blots, see Fig. S1.

### 2.8 Generating GRHL3 overexpressing single‐cell clones

For generating stable GRHL3 single‐cell clones, the cell lines EJ28 and SCaBER were transfected with the *GRHL3*‐pCMV6‐Entry vector or empty vector (OriGene Technologies). 72 h after transfection cells were separated by limiting dilutions, and individual cell clones were selected by incubation in geneticin (G418; EJ28: 1.00 mg·mL^−1^; SCaBER: 1.45 mg·mL^−1^; Thermo Fisher Scientific). Isolated single‐cell clones were verified according to GRHL3 overexpression by real‐time PCR and western blot analyses compared to mock control clones. RNA was isolated from adherent cells using Nucleospin RNA Plus Kit (ANP) (Macherey‐Nagel, Düren, Germany) according to manufacturer's conditions. A second *in vitro* model reflecting urothelial carcinoma was generated by overexpressing GRHL3 (*GRHL3*‐pCMV6‐Entry vector) or empty vector (OriGene Technologies) in invasive J82 cells as stable pools (G418: 0.75 mg·mL^−1^).

### 2.9 Cell growth assay

Cell count assays were used to determine cell growth. 2 × 10^4^ cells per well were seeded into six‐well plates, and living cell number was measured every 24 h for a total of 96 h at 37 °C in 5% CO_2_, using the CASY cell counter (OLS OMNI Life Science, Bremen, Germany).

### 2.10 XTT proliferation assay

Cell growth and proliferation rate of stable GRHL3 and mock clones were analyzed with the XTT cell proliferation kit II (Hoffmann‐La Roche, Basel, Switzerland) according to manufacturer's conditions. The proliferation rate was measured every 24 h for a total of 96 h with ELISA reader Infinite M200 (Bio‐Rad) at absorbance λ = 492 nm.

### 2.11 Colony formation assay

Colony formation was determined as previously described [35] with slight modifications: Cells were plated into six‐well plates (EJ28: 75 × 10^2^ cells per well; SCaBER: 1 × 10^3^ cells per well; J82: 1 × 10^3^ cells per well) in triplicate and cultured in growth medium with G418 for 10 days. After cultivation (37 °C, 5% CO_2_), the colonies were stained in 0.05% crystal violet solution (10% formaldehyde, 80% methanol). Colony formation ability was evaluated semi‐quantitatively using imagej software (National Institutes of Health; Bethesda, MD, USA).

### 2.12 Migration assays

Migration was analyzed by performing a wound healing and Boyden chamber assay. For wound healing, cells were treated with mitomycin C (2.5 μg·mL^−1^; Sigma‐Aldrich, St. Louis, MO, USA) to inhibit cell proliferation [36]. A wound (500 μm) was set with the help of a pipette tip. Cell‐free areas were documented every 8 h for a total of 96 h. Images were captured with an Axiovert 100 TV microscope (Carl Zeiss, Oberkochen, Germany) using cellsens dimension software 2.3 (Olympus, Tokyo, Japan), and the cell‐free areas were quantified with imagej software.

Boyden chamber assays were performed as previously described [37] with slight modifications using 24‐transwell plates with chamber inserts (6.5 mm; Costar, Corning, New York, NY, USA) including a polyethylene terephthalate (PET) membrane (pore size 8.0 μm). Cells were starved for 16 h in serum‐free medium and seeded into the upper chamber of the insert (15 × 10^4^ cells per chamber). Upon chemotactic stimulus with medium containing 10% fetal calf serum (FCS) in the lower chamber, migrated cells were stained in 0.05% crystal violet solution (10% formaldehyde, 80% methanol) and quantified with imagej software.

### 2.13 Cell–matrix adhesion assay

Cell–matrix adhesion was assessed on Matrigel (10 μg·m^−1^, Corning Matrigel Basement Membrane Matrix; Corning) coated plates as previously described [38].

### 2.14 Matrigel invasion assay

To assess cell invasion, 24‐transwell plates with chamber inserts (6.4 mm; Corning BioCoat Matrigel Invasion Chamber), including a PET membrane (pore size 8.0 μm), and coated with Matrigel were used. Cells were starved for 16 h with serum‐free medium, seeded into the upper chamber of the inserts (50 × 10^4^ cells per well), and incubated for 22 h (37 °C, 5% CO_2_). Due to the chemotactic stimulus of medium containing 10% FCS in the lower chamber, cells invade through the porous membrane. The invasion was measured after crystal violet staining (0.05%; 10% formaldehyde, 80% methanol) and quantified with imagej software.

### 2.15 Active RHO detection

Cells were cultured at a density of 1 × 10^5^ cells·cm^−2^ and allowed to attach overnight. After harvest with 1 mm PMSF, lysis buffer cells were pelleted (16 000 ***g***, 15 min, 4 °C). Protein concentrations of cell lysates prepared from supernatants were quantified using a Pierce™ BCA Protein Assay kit (23 225, Thermo Fisher Scientific). RHO kinase activation was measured by using Active RHO Detection Kit (#8820, Cell Signaling) according to the manufacturer's instructions. Provided spin cups were inserted into collection tubes and treated with 100 μL glutathione resin and 400 μg GST‐Rhotekin‐RBP. 500 μg total protein of cell lysis supernatants was added to treated spin cups. GTPγS and GDP were used to provide positive and negative controls appropriately. Reaction mixtures were incubated at 4 °C for 1 h with gentle rocking. After centrifugation (6000 ***g***, 30 s, RT) and three times washing with washing buffer proteins were eluted in 50 μL 2× SDS sample buffer (6000 ***g***, 2 min, RT). The eluted samples were heated for 5 min at 95 °C and were used for SDS electrophoresis.

### 2.16 Immunofluorescence staining

All solutions were prepared in cytoskeleton buffer (CB; EGTA 5 mm, glucose 5 mm, 2‐(*N*‐morpholino)ethanesulfonic acid 10 mm, MgCl_2_ streptomycin 1.72 mm, Sigma‐Aldrich). Samples were incubated with primary antibodies against desmoplakin (Progen, DP‐1), vinculin (Sigma, V9131), and tight junction protein ZO‐1 (ThermoFisher Scientific, 61‐7300). SCaBER and EJ28 cells were cultivated on fibronectin‐coated glass surfaces. For immunocytochemical treatment, cells were fixed with 3.7% paraformaldehyde for 20 min at room temperature (RT). After fixation, the samples were treated with a 30 mm Glycin solution for 10 min at RT, permeabilized with 1% Triton X‐100 for 10 min, and washed thrice with CB. The samples were treated with a blocking solution (0.1% BSA, 0.2% Triton X‐100, 0.05% Tween), containing 5% milk powder for 4 h before incubation with primary antibodies against Desmoplakin (Progen, DP‐1), Vinculin (Sigma, V9131), and ZO‐1 (ThermoFisher Scientific, 61‐7300) diluted 1 : 500 in blocking solution containing 1% milk powder overnight at 4 °C. Treatment with fluorescently coupled secondary antibodies and phalloidin (all ThermoFisher Scientific) was equivalent to primary antibody treatment for 45 min at RT. Samples were washed thrice with CB after each antibody treatment and finally stored in CB at 4 °C in darkness.

Sample treatment with fluorescently coupled secondary antibodies and phalloidin (all Invitrogen, Waltham, MA, USA) was equivalent to primary antibody treatment for 45 min at RT.

Samples were analyzed with the Airy scan detector of an LSM880 (Carl Zeiss) using the ‘Resolution vs. Sensitivity’ mode equipped with a Plan‐Apochromat 63×/1.4 Oil DIC M27 objective (Carl Zeiss). Raw Airy scan image data were processed with the zen black software (Carl Zeiss) in 2D mode. All images of vinculin were acquired focusing on the cell–glass interface, while ZO‐1 and desmoplakin were acquired focusing on the centered cell plane. For statistical analysis of focal adhesions, migrating EJ28 cells contacting less than two cells were imaged. EJ28 cells migrating into the scratch were imaged and analyzed.

### 2.17 Focal adhesion detection

To detect focal adhesions (FA), an in‐house developed python (version 3.8) program was used. In the first step, a cell mask was created to separate the cell from the background. Therefore, the actin and vinculin image channels were summed up into one image, and a threshold was calculated using Li's iterative minimum cross‐entropy method [39, 40, 41]. The threshold was multiplied by 0.7, and all pixels above this threshold were defined as cells. To remove artifacts, only the biggest label within this mask was considered further.

To generate the FA mask, the local z‐score [42] (51 × 51 pixel environment, z‐score threshold = 1) was calculated from the Gaussian smoothed (sigma = 1) vinculin channel. FAs were only considered within the previously created cell mask. Subsequently, FAs with a size of less than 150 pixels were rejected as well as FAs where the mean intensity of the FA was less than the mean intensity of its surrounding pixels.

### 2.18 Next‐generation sequencing and bioinformatic analyses

Total RNA was isolated from cells from different clones as described above. The quantity of RNA was analyzed with the NanoDrop 1000 (Thermo Scientific NanoDrop Technologies). RNA quality control was performed with the Eukaryote Total RNA Nano Series assay using the Agilent 2100 Bioanalyzer system (Agilent Technologies, Inc., Santa Clara, CA, USA). An RNA Integrity Number (RIN) of at least 8 verified the quality of the RNA. Illumina libraries were generated from 1 μg of total RNA using the NEBNext Ultra II Directional mRNA Library Prep Kit for Illumina as described by the manufacturer (New England Biolabs, Frankfurt am Main, Germany). The libraries were run on an Illumina NextSeq500 platform using the High Output v2.5 kit with 150 cycles (Illumina, San Diego, CA, USA). FASTQ files were generated using bcl2fastq (Illumina). Sequencing data were processed using the nf‐core/RNA‐seq pipeline 3.4 [43] with the minimal command. In brief, reads were trimmed by trim galore 0.6.7 [44] and aligned to the Gencode human genome (GRCh38) v28 using star 2.7.9a [45]. Quality assessment was done by PCA plots generated with VST (variance stabilizing transformation) and normalized for all genes but excluded for genes with count lower than 5 in all samples (see Fig. S2). Gene‐level assignment was done using featurecounts 1.6.457 [46]. Transcript‐level quantification was performed by salmon v1.5.2 [47]. The downstream analysis was done using custom scripts in r version 4.1.1. Differential expression analysis was done with custom script with deseq2 package v1.32.0 [48]. Genes with adjusted *P*‐value < 0.05 between the analyzed sample groups were identified as differentially expressed genes (DEG). The NGS data were deposited in the genomics data repository Gene Expression Omnibus (GEO accession: GSE241298). Genome‐wide gene expression profile data were applied in gene set analysis using r package piano_2.8.0 [49]. Curated gene sets for hallmark genes, pathways, and Gene Ontology (Biological Process, Cellular Component, and Molecular Function) in Human msigdb (v2023.1) were gene set collections used in this analysis. The top enriched gene sets were chosen by non‐adjusted *P*‐value with non‐directional option. String‐db [50] was used to visualize putative interaction networks of enriched pathways and biological processes.

### 2.19 Statistical analysis

Statistical analyses were performed using IBM spss 27.0.1.0 (SPSS, Chicago, IL, USA) and graphpad prism 9.0 (GraphPad, San Diego, CA, USA). For comparison of two groups, non‐parametric Mann–Whitney *U* tests were performed. More than two groups were compared with Kruskal–Wallis and Dunn's multiple comparison tests. Spearman correlation coefficients were determined between clinicopathological parameters and *GRHL3* mRNA and protein expression levels. Overall survival (OS) and relapse‐free survival (RFS) rates were analyzed with Kaplan–Meier curves and log‐rank tests. For all tests, two‐sided *P*‐values less than 5% were considered statistically significant.

## 3 Results

### 3.1 Subtype‐specific GRHL3 expression in different histopathological and molecular subtypes of human bladder cancer

**Fig. 1:**
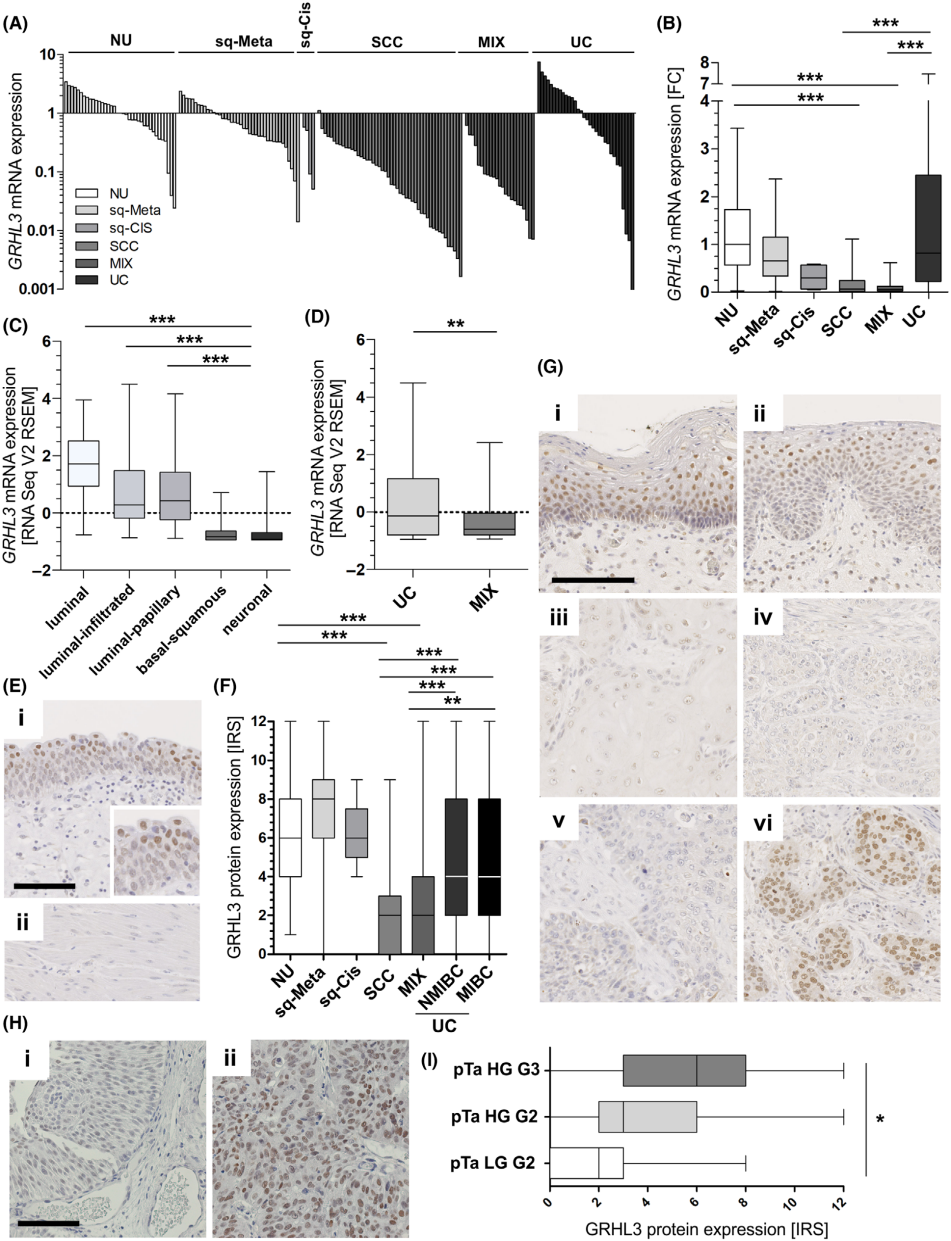
GRHL3 expression in molecular and histological subtypes of bladder cancer. (A, B) *GRHL3* mRNA expression in different histological subtypes of bladder cancer and squamous precursor lesions compared to normal urothelium (NU). (A) Overview of *GRHL3* mRNA expression of overall *n* = 184 samples comprising *n* = 37 NU, *n* = 40 sq‐Meta (squamous metaplasia), *n* = 4 sq‐Cis (squamous carcinoma *in situ*), *n* = 48 SCC (squamous cell carcinoma), *n* = 23 MIX (urothelial carcinoma with squamous differentiation), *n* = 32 UC (urothelial carcinoma). (B) Box plot confirms downregulation of squamous bladder cancers compared to NU and UC ***P < 0.001 (Kruskal–Wallis and Dunn's multiple comparison tests). (C, D) Independent bladder cancers data sets of the TCGA network. (C) *GRHL3* mRNA expression classified by molecular subtypes of human bladder cancer of TCGA. ***P < 0.001 (Kruskal–Wallis and Dunn's multiple comparison tests). (D) Histological subtyping of TCGA data confirmed low *GRHL3* mRNA expression in MIX (*n* = 32) compared to UC (*n* = 73). **P < 0.01 (Mann–Whitney *U* tests). (E–G) GRHL3 protein expression in different histological bladder cancer subtypes and precursor lesions determined by immunohistochemical staining. (E) Strong GRHL3 protein staining in normal urothelium (i) is shown. Highest GRHL3 expression levels were observed in the upper urothelial layer (IRS = 8). Muscle cells and soft tissue (ii) lacked GRHL3 expression and served as staining negative control. Scale bar: 10 μm. (F) Box plot illustrates GRHL3 protein expression for histological subtypes and precursor lesions by using IRS (immunoreactive score) verifying loss of GRHL3 staining in SCC/MIX compared to NU and urothelial NMIBC (non‐muscle‐invasive bladder cancer) and MIBC (muscle‐invasive bladder cancer). **P < 0.01, ***P < 0.001 (Kruskal–Wallis and Dunn's multiple comparison tests). (G) Representative images of immunohistochemically GRHL3 staining of abnormal/diseased tissues: (i) strong GRHL3 protein expression (squamous metaplasia (IRS = 9)), (ii) moderate GRHL3 immunoreactivity (squamous carcinoma in situ (IRS = 6)), (iii) low GRHL3 protein expression (squamous cell carcinoma (IRS = 3)), (iv) minimal protein expression of GRHL3 (squamous cell carcinoma (IRS = 1)), (v) low GRHL3 protein expression (mixed differentiated carcinoma (IRS = 2)), (vi) strong GRHL3 staining (urothelial carcinoma (IRS = 9)). Scale bar: 10 μm. (H) Representative images of immunohistochemically GRHL3 staining of urothelial NMIBC: (i) weak GRHL3 protein expression (pTa low‐grade tumor tissue (IRS = 2)), (ii) strong GRHL3 immunoreactivity (pTa high‐grade G3 tumor cells (IRS = 8)). Scale bar: 10 μm. (I) GRHL3 protein expression of urothelial NMIBC of pTa (papillary, non‐invasive) tumors showing association of loss of GRHL3 staining with histological tumor grade. *P < 0.05 (Kruskal–Wallis and Dunn's multiple comparison tests). For all illustrated box plots: Horizontal lines of box plots: grouped medians. Boxes: 25–75% quartiles. Vertical lines: range, maximum and minimum.

*GRHL3* mRNA expression was studied in a cohort of patient samples (FFPE and fresh‐frozen; overall *n* = 184 samples) comprising normal NU (*n* = 37), squamous metaplasia (*n* = 40), squamous CIS (*n* = 4), pure SCC (*n* = 48), MIX (*n* = 23), and UC (*n* = 32) samples (Fig. 1A,B; for cohort characteristics, see Table S1). Interestingly, *GRHL3* expression differed between cancer subtypes: a significant downregulation was observed in sq‐BLCA. Loss of *GRHL3* mRNA expression was predominant in pure SCC (median fold change (FC) = 0.07; *P* < 0.001) and MIX (median FC = 0.06; *P* < 0.001) compared to the median expression of NU. Squamous precursor lesions of SCC, that is, Sq‐Meta (median FC = 0.66) and Sq‐Cis (median FC = 0.30), already tended to show reduced *GHRL3* mRNA expression. In contrast, urothelial carcinomas remained *GRHL3* mRNA expression (median FC = 0.82) when compared to NU.

Subtype‐specific *GRHL3* mRNA expression was confirmed in an independent bladder cancer data set of The Cancer Genome Atlas (TCGA) (Fig. 1C,D). Histological classification of this cohort into UC (*n* = 270) and MIX (*n* = 46) showed significantly lower *GRHL3* expression in squamous cancers compared to UC (median FC = 0.22; *P* < 0.01; Fig. 1D). Molecular subtyping according to Kamoun et al. [17] confirmed significantly (*P* < 0.001) reduced *GRHL3* mRNA expression in squamous/basal tumors (*n* = 108) compared to luminal‐type bladder cancer including luminal (*n* = 23), luminal‐infiltrated (*n* = 58), and luminal‐papillary (*n* = 110) cancers (Fig. 1C). The neuronal subtype (*n* = 15) also presented significantly (*P* < 0.001) lower *GRHL3* expression levels compared to the luminal subtypes but did not differ from the squamous subtype (Fig. 1C).

GRHL3 expression was further validated on protein level for different histological subtypes. In total, *n* = 503 tissue samples comprising squamous metaplasia (sq‐Meta, *n* = 67), squamous Cis (sq‐Cis, *n* = 5), pure SCC (*n* = 112), UC (*n* = 178), MIX (*n* = 75), and NU (*n* = 66) were immunohistochemically analyzed for GRHL3 (Fig. 1E–G). GRHL3 protein was clearly present in nuclei of NU (median IRS = 7; Fig. 1E (i)). Tissues from sq‐Meta (median IRS = 8; Fig. 1G (i)) and sq‐Cis samples (median IRS = 6; Fig. 1G (ii)) did not show downregulation of GRHL3 protein expression (Fig. 1F). However, in SCC a significant (*P* < 0.001) loss of expression (median IRS = 2; Fig. 1G (iii)) was confirmed. Of note, 41% (42/103) of SCC tissues completely lacked GRHL3 protein expression in nuclei (IRS = 0), whereas 12% (12/103) were characterized by low GRHL3 protein staining (IRS = 3). As expected for a transcription factor, very low amounts of GRHL3 protein were regularly found in the cytoplasm of SCC (18/103). This loss of GRHL3 protein expression was also demonstrated for the MIX phenotype (median IRS = 2; *P* < 0.001; Fig. 1G (v)). In contrast, GRHL3 protein was abundantly (*P* < 0.001) expressed in UC (NMIBC and MIBC) with a median IRS of 4. 14% of UC samples showed increased nuclear GRHL3 protein expression (IRS = 9), and 7% of tissues revealed highest nuclear GRHL3 protein expression (IRS = 12; 13/178; Fig. 1G (vi)). Dividing UCs into NMIBC (*n* = 118) and MIBC (*n* = 60), no difference in nuclear GRHL3 expression between pT stages is representable (*P* = 0.799). However, in NMIBC an increase in nuclear GRHL3 expression is shown between pTa LG G2 (median IRS = 2), pTa HG G2 (median IRS = 3), and pTa HG G3 (median IRS = 6; *P* = 0.019; Fig. 1H,I).

### 3.2 Prognostic impact of GRHL3 expression associated with bladder cancer subtypes

Next, associations of GRHL3 RNA and protein expression with clinicopathological characteristics including tumor stage, histological grading, and nodal status were determined by applying the Fisher exact test in the context of subtype‐specific cohorts. We observed a significant association between increased *GRHL3* mRNA expression and a higher tumor stage in urothelial but not in squamous bladder cancers (Tables S4 and S5). No significant association was shown for GRHL3 protein expression in UC samples to any of the analyzed clinicopathological parameters (Table S6). In turn, for sq‐BLCA we revealed a strong inverse correlation between GRHL3 expression and tumor stage (*P* = 0.036, *r* = −0.258; Table 1). Pretreatment with neoadjuvant chemotherapy (NAC) showed no association with GRHL3 expression (Table 1 and Tables S4–S6). Correlating clinicopathological parameters and nuclear GRHL3 protein expression in NMIBC, a strong positive association with grading (*P* = 0.008, *r* = 0.268) and gender (*P* = 0.004, *r* = 0.320) was found (Table S7). This correlation of grading and nuclear and cytoplasmic GRHL3 protein expression was proven over all UCs (NMIBC and MIBC; HG/LG: *P* = 0.021, *r* = 0.238; Table S8).

**Table 1:** Clinicopathological parameters in relation to GRHL3 expression of sq‐BLCA in patient cohort. Significant *P*‐values are marked in boldface.

	GRHL3 expression IRS^a^				
	*n* ^b^	Low	High	*p*‐value^c^	Spearman *r*
Parameter					
Gender					
Female	45	14	31	0.254	−0.115
Male	21	9	12		
Tumor stage^d^					
pT1‐pT2	34	9	25	**0.036**	−0.285
pT3‐pT4	20	11	9		
Grading^d^					
G2	27	6	11	0.586	−0.012
G3	41	15	26		
pN status^d^					
pN negative	15	8	7	0.477	−0.122
pN positive	6	4	2		
NAC^e^					
No	30	14	16	0.244	0.271
Yes	3	0	3		

^a^ Dichotomized at 25% quartile.

^b^ Only patients with primary bladder cancer were included.

^c^ Fisher's exact test.

^d^ According to WHO 2004 classification.

^e^ Neoadjuvant chemotherapy (NAC).

**Fig. 2:**
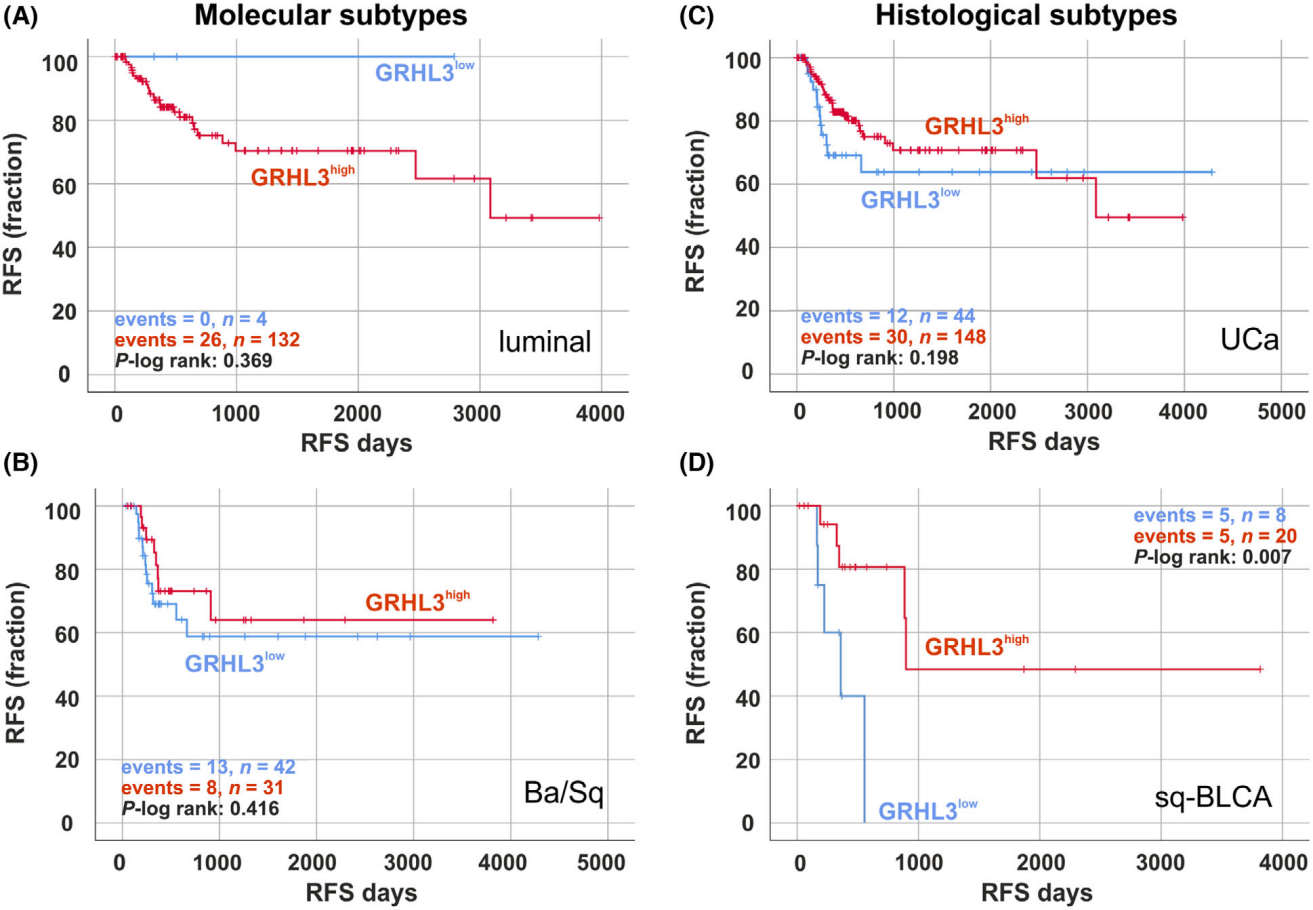
*GRHL3* mRNA expression predicts longer RFS in bladder cancer. (A–D) Kaplan–Meier survival curves illustrate relapse‐free survival (RFS) of bladder cancer patients with high GRHL3 mRNA expression (red curve) compared to low GRHL3 mRNA expression (blue curve) classified by molecular subtypes, that is, luminal type (A), basal/squamous‐like (Ba/Sq) (B), and histological subtypes, that is, urothelial carcinomas (UCa) (C), and squamous bladder cancer (Sq‐BLCA) (D) based on TCGA BLCA data sets.

Additionally, we used TCGA data sets to determine patient outcome by calculating univariate Kaplan–Meier survival curves to classify into histological and molecular subtypes. Since *GRHL3* is weakly expressed in basal/squamous (BASQ) tumors, we dichotomized expression levels into low and moderate to strong *GRHL3* expression by considering the lower 25% quartile to define the cut‐off. Based on that, relapse‐free survival (RFS) rate of patients diagnosed with BASQ UC tended to a slight longer RFS in the setting of increased *GRHL3* expression (Fig. 2) but without significance. Significance was also missed for luminal UC; however, none of the luminal bladder tumors characterized by low *GRHL3* mRNA expression presented an event. Classifying this data set by histological parameters, we demonstrated for patients with sq‐BLCA that RFS was significantly (*P* = 0.007) longer with medium to strong *GRHL3* expression (mean RFS: 2191.4 days ±568.8, 95% CI 1076.6–3306.3) compared to tumors with low *GRHL3* expression (mean RFS: 368.9 days ±70.8, 95% CI 230.1–507.7) (*n* = 28; Fig. 2).

### 3.3 GRHL3 mediates subtype‐specific growth of bladder cancer cells and colony forming

**Fig. 3:**
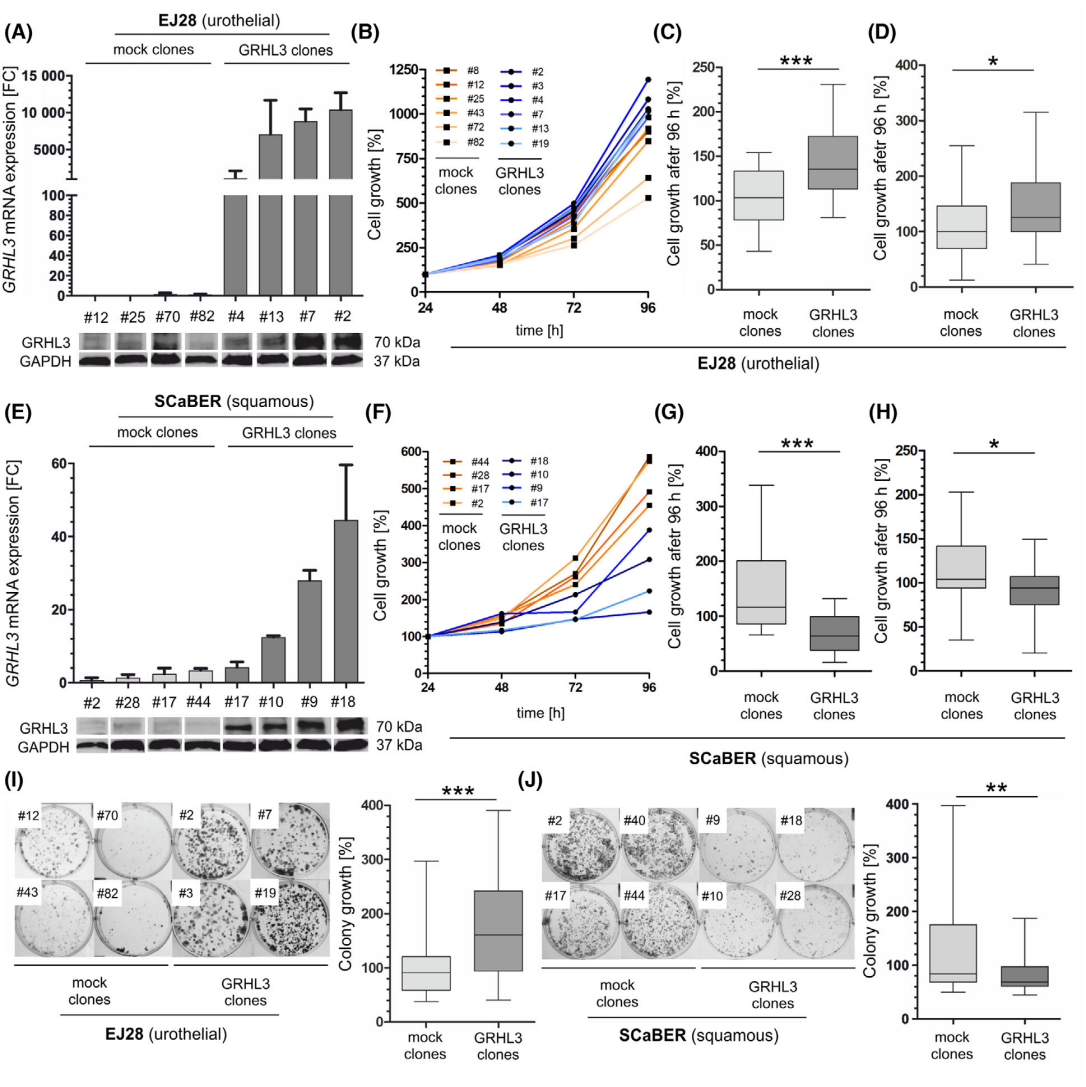
GRHL3 overexpression affects tumor cell growth and colony formation depending on different bladder cancer cell lines. (A) GRHL3 overexpression of EJ28 cell line. Top: Relative GRHL3 mRNA expression based on real‐time PCR comparing mock clones and stable GRHL3 clones. Vertical lines: +SEM. Bottom: Immunoblotting confirms GRHL3 (70 kDa) protein expression in stable GRHL3 clones as compared to mock clones. GAPDH expression served as loading control (37 kDa). (B) Cell count assay (*n* = 4 independent experiments). EJ28 GRHL3 clones (*n* = 6) show markedly increased cell growth in comparison to EJ28 mock clones (*n* = 6). (C) Box plot analyses of cell growth (*n* = 4 independent experiments) after 96 h comparing EJ28 mock clones (*n* = 6) and EJ28 GRHL3 clones (*n* = 6). ***P < 0.001 (Mann–Whitney *U* tests). (D) XTT proliferation assay (*n* = 5 independent experiments). Box plot analyses of cell proliferation after 96 h prove significantly increased proliferation rates in EJ28 GRHL3 clones (*n* = 6) compared with EJ28 mock clones (*n* = 6). *P < 0.05 (Mann–Whitney *U* tests). (E) GRHL3 overexpression of SCaBER cell line. Top: Relative GRHL3 mRNA expression based on real‐time PCR comparing mock clones and stable GRHL3 clones. Vertical lines: +SEM. Bottom: Immunoblotting confirms GRHL3 (70 kDa) protein expression in stable GRHL3 clones as compared to mock clones. GAPDH expression served as loading control (37 kDa). (F) Cell count assay (*n* = 4 independent experiments). SCaBER GRHL3 clones (*n* = 4) show markedly decreased cell growth compared to SCaBER mock clones (*n* = 4). (G) Box plot analyses of relative cell numbers (*n* = 4 independent experiments) after 96 h reveal significantly reduced cell growth of SCaBER GRHL3 clones (*n* = 4) compared to SCaBER mock clones (*n* = 4). ***P < 0.001 (Mann–Whitney *U* tests). (H) XTT proliferation assay (*n* = 5 independent experiments). Box plot analyses demonstrate significantly decreased cell proliferation after 96 h comparing SCaBER GRHL3 clones (*n* = 4) with SCaBER mock clones (*n* = 5). *P < 0.05 (Mann–Whitney *U* tests). (I) Colony formation assay (*n* = 5 independent experiments). Left: Representative colony formation assay of EJ28 mock clones (#12, #43, #70, #82) and GRHL3 clones (#2, #3, #7, #19). Right: Box plot analyses of colony formation of EJ28 GRHL3 clones (*n* = 6) compared to mock clones (*n* = 6). ***P < 0.001 (Mann–Whitney *U* tests). (J) Colony formation assay (*n* = 4 independent experiments). Left: Representative colony formation assay of SCaBER mock clones (#2, #17, #40, #44) and GRHL3 clones (#9, #10, #18, #28). Right: Box plot analyses of colony formation of SCaBER GRHL3 clones (*n* = 4) compared to mock clones (*n* = 4). **P < 0.01 (Mann–Whitney *U* tests). For all illustrated box plots: Horizontal lines: grouped medians. Boxes: 25–75% quartiles. Vertical lines: range, maximum and minimum.

Since a reverse GRHL3 expression was revealed for squamous and urothelial bladder cancers, we aimed to analyze the functional role of GRHL3 in different bladder cancer subtypes *in vitro*. We stably overexpressed GRHL3 in urothelial EJ28 and the basal/squamous SCaBER cell lines and extended with a second model for invasive urothelial carcinomas by overexpressing GRHL3 in urothelial stable J82 pools. Overexpression of GRHL3 in EJ28 cells verified by RT‐qPCR and western blot analyses (Fig. 3A) caused a significantly (*P* < 0.001) increased cell growth for 96 h when compared to mock controls (median growth: +32%) (Fig. 3B,C). These findings were confirmed by independent XTT assays demonstrating increased cell proliferation of GRHL3 overexpressing clones (median cell growth: +26.2%, *P* < 0.05) compared to controls (Fig. 3D). In turn, GRHL3 overexpression (Fig. 3E) caused impaired cell growth (median growth: −52.6%, *P* < 0.001) in SCaBER‐derived clones compared to mock controls (Fig. 3F,G). Blocked cell growth by GRHL3 was confirmed by XTT assays (median growth: −9.6%, *P* < 0.05; Fig. 3H).

In addition, we revealed a markedly increased colony formation of urothelial EJ28 cells when overexpressing GRHL3 (median colony growth: +71.6%, *P* < 0.001) (Fig. 3I). Distinctly increased colony formation was likewise shown in urothelial J82 cells overexpressing GRHL3 (median colony growth: +26.8%, *P* < 0.001) (Fig. S3). In SCaBER, GRHL3 overexpression led to decreased colony formation as compared to mock clones (Fig. 3J). The densitometric analysis of relative colony growth confirmed a significant (*P* < 0.01) colony growth inhibition of up to 69%.

### 3.4 GRHL3 affects motility and invasion of bladder cancer cells depending on the cancer subtype

**Fig. 4:**
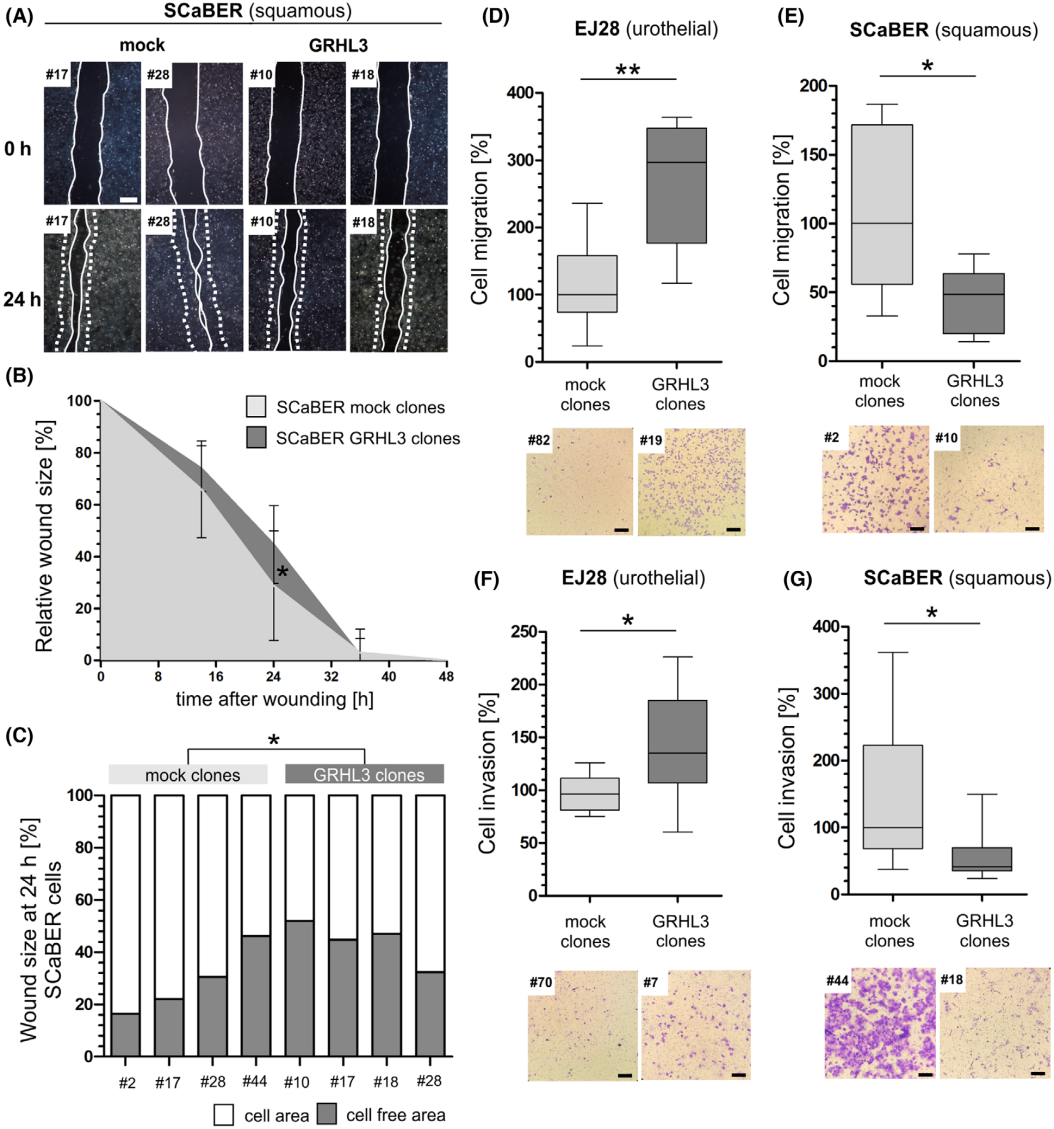
GRHL3 overexpression alters cell migration and invasion depending on the subtype of bladder cancer cell lines. (A–C) Wound healing assay (*n* = 3 independent experiments). (A) Representative pictures of the scratch at 24 h comparing SCaBER mock clones (#28, #17) with SCaBER GRHL3 clones (#10, #18). Dotted lines: Original wound size, solid lines: effective wound size *t* = 24 h. (B) SCaBER GRHL3 clones (*n* = 4) display suppressed cell migration ability comparing the relative wound size with SCaBER mock clones (*n* = 4) over 48 h. *P < 0.05 (Kruskal–Wallis and Dunn's multiple comparison tests). Vertical lines: standard deviation (SD). (C) Detailed analyses of relative wound size at 24 h comparing individual SCaBER mock and GRHL3 clones confirming a significantly increased wound size of SCaBER GRHL3 clones. *P < 0.05 (Kruskal–Wallis and Dunn's multiple comparison tests). (D, E) Boyden chamber migration assay (*n* = 4 independent experiments). (D) Box plot analysis of migration assays represents the increased capacity of cell migration of EJ28 GRHL3 clones (*n* = 2) as compared to EJ28 mock clones (*n* = 2). **P < 0.01 (Mann–Whitney *U* tests). (E) Box plot analyses of SCaBER cell migration potential reveal significantly lower motility of SCaBER stable GRHL3 clones (*n* = 2) in comparison to SCaBER mock clones (*n* = 2). *P < 0.05 (Mann–Whitney *U* tests). Bottom: Representative pictures of Boyden chamber assay for EJ28 and SCaBER clones (Scale bar: 200 μm). (F, G) Invasion assay (*n* = 4 independent experiments). (F) Box plot analyses of the capacity of cell invasion show a significantly higher number of invasive cells of EJ28 GRHL3 clones (*n* = 3) compared to EJ28 mock clones (*n* = 3). *P < 0.05 (Mann–Whitney *U* tests). (G) Box plot analyses of invasion assays showed significantly reduced invasion of SCaBER stable GRHL3 clones (*n* = 3) compared with SCaBER mock clones (*n* = 3). *P < 0.05 (Mann–Whitney *U* tests). Bottom: Pictures of invaded cells representatively illustrated for SCaBER and EJ28 clones (Scale bar: 200 μm). For all illustrated box plots: Horizontal lines: grouped medians. Boxes: 25–75% quartiles. Vertical lines: range, maximum and minimum.

Performing a wound healing assay for both models, we further demonstrated an effect on cell motility of GRHL3 SCaBER clones compared to mock controls (Fig. 4A): The wound size at 24 h of SCaBER GRHL3 clones closed 45% in mean of the original scratch size, while mock clones were already able to repopulate 71% of the wounded area (*P* < 0.05; Fig. 4B,C). For EJ28 cells, we did not observe any significant impact of GRHL3 on wound closure (data not shown). However, based on transwell assays allowing a specific documentation of cell migration without any unspecific cell growth effects, an increased migration capacity of GRHL3 overexpressing EJ28 clones was shown compared to mock controls (median cell motility: +196%, *P* < 0.01; Fig. 4D). Contrary to that, GRHL3 overexpression in SCaBER cells was characterized by reduced motility (median cell motility: −52%, *P* < 0.05) as compared to controls (Fig. 4E) confirming the wound healing data.

Finally, we analyzed the impact of GRHL3 overexpressing cells to invade through a simulated basal membrane using Matrigel‐coated transwell assays. In EJ28, GRHL3 expression fostered the invasion potential (median invading cells: +39%; Fig. 4F), while causing a lower ability of cell invasion (median invading cells: −59%) in SCaBER cells compared to mock clones (Fig. 4G).

### 3.5 GRHL3 modulates cell–matrix interactions and RHO activity

Next, transcriptomic analyses of independent GRHL3 overexpressing SCaBER (*n* = 3) and EJ28 (*n* = 2) clones compared to corresponding independent mock clones (for each model *n* = 3) were performed. Gene set enrichment analysis was used to gain insights into GRHL3‐affected hallmarks, pathways, and gene ontology (GO), that is, biological processes (BP), cellular compartments (CC), and molecular functions (MF) in context of used cell lines. Top 15 enrichment results for each category and cell lines are listed in Table S9. Overall, we observed enrichment of genes associated with epithelial‐to‐mesenchymal transition (EMT) (hallmarks), integrin complexation (CC), collagen receptor activity (MF), and integrin‐mediated cell adhesion (BP). There were also cell line‐specific enrichments: In SCaBER, we observed enrichment of sets of genes involved in actin cytoskeleton and the myosin complex (MF and CC). Heatmaps and associated interaction networks for integrin‐mediated cell adhesion in EJ28 and actin cytoskeleton in SCaBER are visualized in Fig. S4. In EJ28 cells, chromatin binding and RNA polymerase II transcription regulatory (MF and CC), among others, were enriched upon GRHL3 expression.

**Fig. 5:**
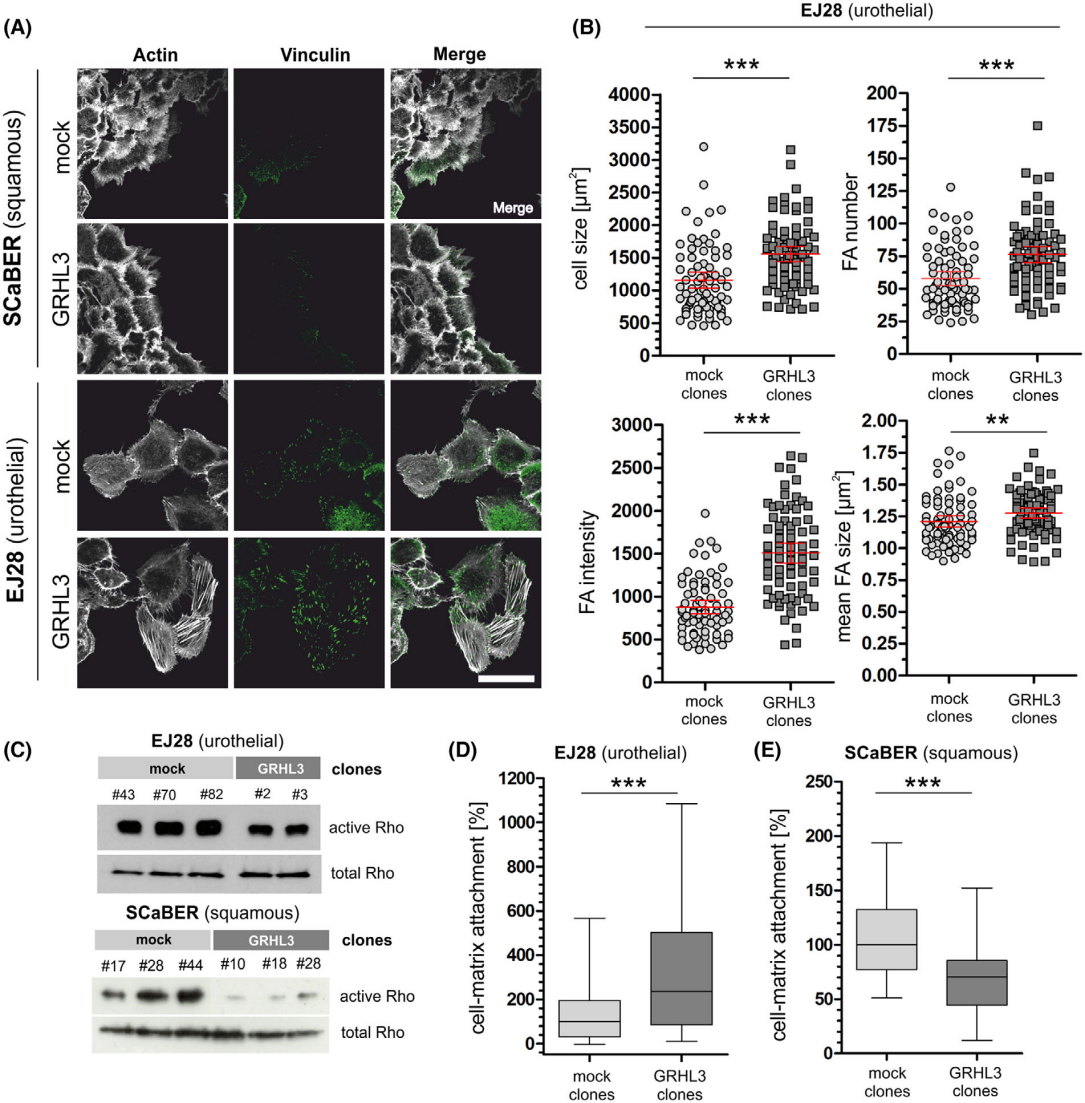
GRHL3 modulates cell–matrix interactions and intracellular signaling. SCaBER and EJ28 cells adhere to substrate via focal adhesions. (A) Confocal images of representative GRHL3 overexpressing clones (overall *n* = 2, illustrated clones: #18 (SCaBER) and #2 (EJ28)) and corresponding mock clones (overall *n* = 2, illustrated clones: #28 (SCaBER) and #43 (EJ28)) show the actin cytoskeleton (phalloidin, white), focal adhesions (FA) (Vinculin, green) of cells adjacent to a scratch wound introduced to a confluent cell layer 16 h prior to fixation. Image contrast was adapted for the accurate representation of FA presence in both cell lines and does not represent relative vinculin signal intensity. Scale bar = 50 μm. (B) Statistical analysis of FA intensity, mean size, and number as well as cell size in EJ28 cells migrating into the scratch wound shown in A before fixation. Scatter plots show the respective values calculated for GRHL3‐expressing (*n* = 77) and mock cells (*n* = 77) from vinculin signal. Bars show the median (red) and 95% confidence interval (black). **P < 0.01; ***P < 0.001 (Mann–Whitney *U* tests). (C) Analysis of integrin downstream signaling. Representative western blot results illustrate activated RHOA GTPases in independent GRHL3‐expressing EJ28 and SCaBER clones compared to mock clones. Total RHOA served as loading control. (D, E) Cell adhesion assay (*n* = 4 independent experiments). (D) Box plot analyses of cell–matrix adhesion illustrate a significantly higher relative number of adhesive cells of EJ28 GRHL3 clones (*n* = 4) as compared to EJ28 mock clones (*n* = 4). ***P < 0.001 (Mann–Whitney *U* tests). (E) Box plot analyses of cell adhesion of SCaBER GRHL3 clones (*n* = 4) show significantly reduced cell adhesion compared with SCaBER mock clones (*n* = 4). ***P < 0.001 (Mann–Whitney *U* tests). For all illustrated box plots: Horizontal lines: Grouped medians. Boxes: 25–75% Quartiles. Vertical lines: Range, maximum and minimum.

Since integrin‐mediated cell adhesion might be affected by GRHL3, we next analyzed focal adhesion‐mediated cell–matrix coupling (marker: vinculin), cell migration‐related actin cytoskeleton organization, and cell–cell adhesion complexes (tight junction‐specific marker: zonula occludens 1 (ZO‐1) and desmosome‐specific marker: desmoplakin). EJ28 and SCaBER clones were immunofluorescently stained against the marker proteins and analyzed 16 h after wounding confluent monolayers (Fig. 5A and Figs S5 and S6).

Typical characteristics of the subtype‐specific cell identity were observed in cell lines mirrored by tight growth patterns with pronounced cell–cell coupling by desmosomes and tight junctions in squamous‐like SCaBER cells. Urothelial EJ28 cancer cells showed only weak intercellular coupling indicated via sparse tight junction and desmosome formation at distinct contact sites (Fig. S6). At the edges of the scratch mesenchymal‐like single‐EJ28 cells rich in contractile actin stress fibers migrated into the wounded area (Fig. S5). In contrast, the collective cell migration of SCaBER clones was accompanied by reduced stress actin fiber formation. SCaBER cells exhibited cytoskeletons with pronounced cortical actin structures. Both phenotypes were found to be independent of GRHL3 expression. However, calculating the number and size of FA sites demonstrated a clear regulation of FAs in GRHL3‐expressing EJ28 clones, which correlate with enlarged cell sizes (Fig. 5B). The significant increase in focal adhesion number, size, and vinculin intensity reflected GRHL3‐induced cell–matrix adhesion dynamics. The larger cell size (Fig. 5B) and the actin stress fiber‐rich phenotype demonstrated GRHL3‐dependent cell spreading and migration during wound healing in EJ28 cancer cells. This finding further suggested that the observed shift in FA dynamics is linked with enhanced contractile cell force in these urothelial cancer cells. So we analyzed RHO GTPase dynamics as primary mediators for actomyosin force‐based cell motility using a pulldown assay. Indeed, we observed a nearly complete loss of RHOA GTPase activity in SCaBER clones overexpressing GRHL3 compared to mock control clones, indicating a complete imbalance of RHO‐RAC mediated signaling cascade. In contrast, GRHL3‐expressing EJ28 clones showed only a slight reduction in RHOA activity compared to controls (Fig. 5C), suggesting a functional and putative dynamic RHO‐RAC turnover [51].

Finally, we studied the influence of GRHL3 expression on cell–matrix adhesion capability of our *in vitro* models. An increased adhesion on Matrigel of GRHL3‐expressing EJ28 cells (median + 135%, *P* < 0.001) was observed compared to mock clones (Fig. 5D). This result is consistent with the previously observed increase in focal adhesion formation (Fig. 5B). In turn, SCaBER cells overexpressing GRHL3 were characterized by impaired cell–matrix adhesion, that is, GRHL3 SCaBER clones showed significantly (*P* < 0.001) decreased attachment to Matrigel by 29% in median (Fig. 5E).

### 3.6 Transcriptomic profiling reveals GRHL3‐specific gene patterns and regulation of EIF4E3 in dependence on the cell line model

**Fig. 6:**
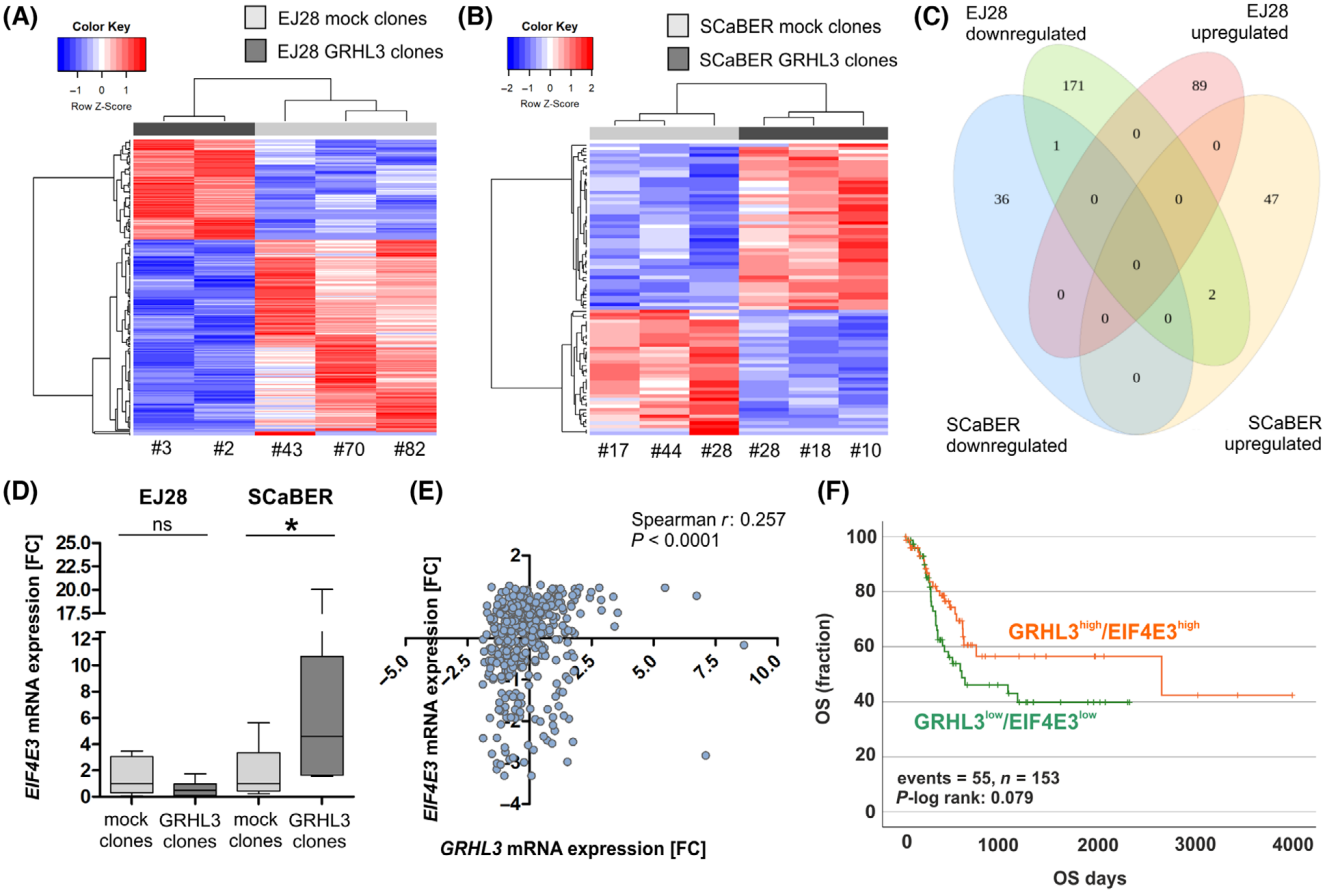
Downstream consequences of GRHL3 reveal subtype‐specific gene expression patterns. (A, B) Heatmaps illustrate GRHL3‐associated differentially expressed gene sets (DEGs) (*P*adj < 0.05) for EJ28 (*n* = 5) and SCaBER (*n* = 6) clones. Comparison of gene expression profiles is based on independent GRHL3‐expressing and mock single‐cell clones (red: upregulated; blue: downregulated). (C) Venn diagram illustrates exclusive and overlapping genes of the EJ28 and SCBER clone DEGs. (D) Confirming co‐ and inverse regulation between *GRHL3* and *EIF4E3* mRNA expressions in EJ28 and SCaBER clones expressing GRHL3 (each *n* = 5) compared to control clones (each *n* = 4) using RT‐qPCR. *P < 0.05 (Mann–Whitney *U* tests). (E) Spearman correlation analyses illustrate significant co‐expression of GRHL3 and EIF4E3 mRNA expressions in bladder cancer (TCGA data set). (F) GRHL3‐EIF4E3 expression axis tends to predict favorable prognosis of bladder cancer patients. Kaplan–Meier survival curves illustrate overall survival (OS) of patients with high *GRHL3‐EIF4E3* mRNA expression (orange curve) compared to low *GRHL3‐EIF4E3* mRNA expression (green curve) based on TCGA BLCA data sets. For all illustrated box plots: Horizontal lines: grouped medians. Boxes: 25–75% quartiles. Vertical lines: range, maximum and minimum.

Based on RNA‐seq profiling data, we aimed to decipher subtype‐specific gene patterns and putative target genes of *GRHL3* in SCaBER and EJ28 tumor cells and identified *GRHL3*‐regulated differential expressed gene (DEG) sets (adjusted *P* ≤ 0.05) comprising *n* = 263 genes in EJ28 and *n* = 86 genes in SCaBER clones (Fig. 6A,B and Tables S10 and S11). Overall, *n* = 174 genes were inversely regulated, and *n* = 69 genes were induced in *GRHL3*‐expressing EJ28 clones. In SCaBER clones, *n* = 37 genes were repressed and *n* = 49 genes were co‐expressed with *GRHL3*. Principal component analysis of RNA‐seq data is shown in Fig. S2. Downregulation of *RHOG*, a potential target gene of GRHL2 in non‐small lung cancer [52], was observed in GRHL3‐expressing SCaBER cells. Inverse regulation of *GRHL3* and *RHOG* was confirmed by RT‐qPCR using independent GRHL3‐expressing SCaBER single‐cell clones compared with mock clones (Fig. S7). Interestingly, only a small overlap of three genes (0.9%: *TMEM98*, *EIF4E3*, *BMF*) between both cell lines was observed (Fig. 6C). Of these genes, only *TMEM98* was similarly expressed in both models, whereas both *EIF4E3* and *BMF* have been identified as potentially reversely regulated (Fig. 6D). *EIF4E3* was confirmed to be upregulated in a set of *n* = 5 independent SCaBER single‐cell clones overexpressing *GRHL3* as compared to *n* = 4 independent mock clones, whereas downregulation missed significance in EJ28 *GRHL3* clones (Fig. 6D).

A putative binding site for *GRHL3* was found in intron 5 (GAAA**C**CA**G**CCTGA**C**AG**G**ATTG) of the *EIF4E3* gene comprising the characteristic GRHL binding motifs, that is, two adjacent repeats of Grainyhead consensus sequences with two tandem core CNNG motifs set apart by five bases [5]. Co‐regulation of *GRHL3* and *EIF4E3*, a putative tumor suppressor [53], was then verified by Spearman rank correlation in the TCGA BLCA data set (Spearman *r* = 0.257, *P* < 0.001, Fig. 6E). Of clinical relevance, increased expression of the *GRHL3‐EIF4E3* axis tends to predict prolonged overall survival of patients with bladder cancer as compared to those with low expression of *GRHL3‐EIF4E3*, pointing to a pathophysiological impact of this co‐regulation in bladder cancer (Fig. 6F).

## 4 Discussion

In the present study, we show evidence for a dual role of GRHL3 in the context of histological and molecular subtypes of cancers arising from the urothelium. Our comprehensive expression study provides insights into subtype‐specific *GRHL3* expression associated with different clinicopathological associations and patients' outcomes. Previously, Wezel et al. [22] provided insight into the potential role of GRHL3 in urothelial carcinomas, with a special focus on urothelial growth patterns focusing on a papillary and invasive urothelial model. They also showed that luminal non‐invasive RT4 tumor cells seem more aggressive when downregulating GRHL3, while overexpression slightly impaired cell migration and invasion in T24 cells [22], hence postulating a generally tumor‐suppressive function of GRHL3 in urothelial carcinomas (UC). However, GRHL3 expression was determined in cell lines only. Here, we determined GRHL3 mRNA and protein expression considering more than 600 human tissue samples and 314 TCGA samples identifying high GRHL3 mRNA and protein levels associated with increased tumor stage and a trend toward shorter recurrence‐free survival in UC. We demonstrated that loss of GRHL3 expression was abundant and associated with increased tumor stage and unfavorable prognosis in squamous bladder cancers. The correlation of GRHL3 and tumor stage was previously shown by Yuan et al. [54] in colorectal cancer. In breast cancer, Xu et al. [10] reported subtype‐specific expression of GRHL3 with lower levels in triple‐negative breast cancer, and persistent GRHL3 expression was generally associated with longer overall survival of patients with lymph node metastases.

Consistent with these findings, our *in vitro* models provide functional evidence that *GRHL3* is closely associated with a distinct molecular background of cells depending on histological differentiation. Overexpression in urothelial EJ28 and J82 cells drives oncogenic features, whereas squamous‐differentiated SCaBER cells cause opposite, that is, tumor‐suppressive, functions mediated by GRHL3. In fact, GRHL3 expression fostered cell growth, colony formation, cell migration, and invasion in EJ28 clones and increased colony formation in J82 cells. Contrary to that, GRHL3 overexpressing SCaBER clones were characterized by impaired cell growth and effectively suppressed migration, invasion, and the clonogenic potential. So far, opposed roles of grainyhead‐like proteins have been shown between various tumor entities [6], and suppressive functions have been often observed in squamous‐like cancer types. Loss of GRHL3 expression was first described in SCC of the skin [11, 55] associated with tumor‐suppressive properties. In head and neck SCC, Saffarzadeh et al. [56] also demonstrated downregulation of GRHL3, whereas oncogenic properties of *GRHL3* were described in colorectal cancer [13, 54] and diffuse large B‐cell lymphoma [14].

A rationale for the divergent role of the *GRHL* gene family might be its function as a pioneer transcription factor, directly binding to chromatin, especially regulating epithelial gene expression. Klein et al. [57] observed specific binding of *GRHL3* at distinct enhancers and promotors depending on the differentiation status of keratinocytes. They demonstrated that the chromatin relocates *GRHL3* binding and enhancers to regulate the irreversible commitment of progenitor keratinocytes to differentiation and a reversible transition to migration. Thus, accessible DNA regions in individual cells may coin the downstream consequences of GRHL3 activity as observed in our urothelial EJ28 and squamous SCaBER model. This context‐specific regulation of gene sets by GRHL3 is mirrored by a marginal overlap of significant gene expression patterns between our models. Interestingly, affected biological processes showed greater similarity, whereas underlying sets of enriched genes mostly vary, which might be a basis for the reversely functional outcome of GRHL3 expression in the urothelial and squamous *in vitro* model. In both lines, GO analyses suggest that epithelial‐to‐mesenchymal transition (EMT) might be affected, which is consistent with previous studies potentially each with a different outcome for cell behavior. During the progression of various tumor entities, GRHL transcription factors appear to mediate the broad spectrum of involved players of the EMT process modulating EMT/MET dynamics [58]. A decisive role of GRHL3 in tumor development is also attributed to cell adhesion [59] as demonstrated in our bladder cancer models. Overall, we observed changes in cell–matrix interactions, both functionally and on the expression level of distinct surface receptors such as integrins confirmed by the modulated assembly of FA sites.

In single‐migrating EJ28 cells, GRHL3 re‐expression increased with the FA size and number. This finding emphasizes an impact of GRHL3 on cell–matrix adhesion‐mediated motility, similar to other studies dealing with FA and showed that size predicts migration speed, for instance, in highly invasive fibrosarcoma cells [60]. This notion was functionally supported by increased cell migration, cell–matrix adhesion, and invasiveness of GRHL3‐expressing EJ28 clones. The impact of GRHL3 on wound healing and cell migration was first described in epidermis, and modulated cell adhesion potential of tumor cells triggered by GRHL3 was demonstrated [61, 62, 63]. *ARHGEF19* (RhoGEF19) was previously identified as a target gene of *GRHL2* and *GRHL3* affecting the maintenance of epidermal differentiation by activating RHOA and regulating the planar cell polarity signaling pathway in epidermal wound repair [63, 64]. Consistent with these findings, we revealed modulated RHO GTPase activity in bladder cancer cells. In EJ28 cells, slight effects on RHOA activity could be the outcome of a dynamic turnover between RHO and RAC signaling causative for higher cellular motility, as mesenchymal migration is characterized by RAC activity at the leading edge, whereas RHO is active toward the cell rear, together resulting in a lamellipodium at the leading edge [65]. In turn, activity loss of RHOA in SCaBER cells suggests a substantial imbalance of underlying processes. However, squamous SCaBER cells form generally less actin stress fibers associated with collective cell migration capacities. Hence, the impact of retarded RHO activation on cytoskeleton and FA remodeling remains unclear. Since RHO GTPases are known to control various processes like cell–cell adhesion, vesicle trafficking, cell cycle, or collective cell polarization [66], implications of RHO activity loss caused by GRHL3 expression in squamous cancer cells might be multifactorial and should be further addressed in future studies.

## 5 Conclusion

In conclusion, our comprehensive expression data of GRHL3 in different bladder cancer subtypes are associated with distinct prognostic implications. The functional outcomes *in vitro* argue for a histological subtype‐specific impact of GRHL3, that is, a tumor‐suppressive effect of GRHL3 in sq‐BLCA and, in contrast, a more oncogenic property in urothelial carcinomas with a putative role of the *GRHL3‐EIF4E3* expression axis involving integrin and actin‐associated processes and pathways and factors like *ARHGEF19*. Since *ARHGEF19* (RhoGEF19) is known to activate RHOA [62], while RHOE has been postulated as antagonist of RHOA [67] and affects EIF4E family members by impairing their cap‐dependent translational functions [68], a putative interdependent feedback between these is conceivable but remains speculative at this stage. Bearing in mind that Wezel et al. observed tumor‐suppressive effects in urothelial T24 cells, the configuration of the molecular context such as chromatin structures might be finally crucial for GRHL3 function. Thus, our study along with prior research efforts gain further insights into role of GRHL3 transcription factor helping to further decipher the clinically important pathways of bladder cancer subtypes.

## Conflict of interest

The authors declare no conflict of interest.

## Author contributions

MR, NTG, and EN were involved in conception and design. FCL and JW performed expression data and conducted *in vitro* models. FCL, JP, and JW were involved in functional experiments. FCL, SL, TS, and RK were involved in collecting cohorts and immunohistochemical analyses. EN, FF, and YH carried out immunofluorescence analyses. LG performed RNA‐seq and analyzed data. MR, NTG, RW, JM, and DDJ discussed and/or supervised the project. FCL wrote the original draft. MR, NTG, EN, FF, and LG edited the manuscript. All authors critically read, reviewed, and agreed to the submitted version of the manuscript.

### Peer review

The peer review history for this article is available at https://www.webofscience.com/api/gateway/wos/peer‐review/10.1002/1878‐0261.13623.

## Supporting information


**Fig. S1.** Raw data and uncropped images of western blots presented in Figs 3, 5, and Fig. S3.
**Fig. S2.** Principal component analysis (PCA) of transcriptomic data sets.
**Fig. S3.** GRHL3 overexpression affects colony formation in urothelial J82 cancer cells.
**Fig. S4.** Visualization of enrichment of gene sets involved in integrin complexation in urothelial EJ28 (A, B) and in actin cytoskeleton in SCaBER clones (C, D).
**Fig. S5.** Epithelial cell–matrix adhesion pattern in GRHL3‐expressing SCaBER and EJ28 cells.
**Fig. S6.** Epithelial cell–cell adhesion pattern in GRHL3‐expressing SCaBER and EJ28 cells.
**Fig. S7.** GRHL3 causes downregulation of RHOG in squamous bladder cancer cells.
**Table S1.** Clinicopathological parameters of patients with urinary bladder cancer (*n* = 264) of the archive of the Institute of Pathology RWTH Aachen analyzed in this study.
**Table S2.** Clinicopathological parameters of patients with non‐muscle‐invasive bladder cancer (NMIBC; *n* = 107 cases, *n* = 46 patients) analyzed in this study.
**Table S3.** Primer sequences and PCR conditions.
**Table S3.1.** Primer sequences for RNA analyses.
**Table S3.2.** Mastermix for qPCR.
**Table S3.3.** Cycle conditions of qPCR.
**Table S3.4.** Mastermix for cDNA synthesis.
**Table S3.5.** Cycle conditions of cDNA synthesis.
**Table S4.** Clinicopathological parameters in relation to *GRHL3* expression of UC in patient cohort.
**Table S5.** Clinicopathological parameters in relation to *GRHL3* expression of sq‐BLCA in patient cohort.
**Table S6.** Clinicopathological parameters in relation to GRHL3 expression of UC in patient cohort.
**Table S7.** Clinicopathological parameters in relation to GRHL3 expression of NMIBC in patient cohort.
**Table S8.** Clinicopathological parameters in relation to GRHL3 expression of UC in patient cohort (NMIBC and MIBC).
**Table S9.** Gene set enrichment analyses of GRHL3‐expressing clones.
**Table S10.** GRHL3 regulated differential expressed gene (DEG) set (adjusted *P* ≤ 0.05) identified in EJ28 clones.
**Table S11.** GRHL3 regulated differential expressed gene (DEG) set (adjusted *P* ≤ 0.05) identified in SCaBER clones.

## Data Availability

The data that support the findings of this study are openly available in genomics data repository Gene Expression Omnibus (GEO accession: GSE241298; reviewer access token: wjafkmacpjybxqh): https://eur02.safelinks.protection.outlook.com/?url=https%3A%2F%2Fwww.ncbi.nlm.nih.gov%2Fgeo%2Fquery%2Facc.cgi%3Facc%3DGSE241298&data=05%7C01%7C%7C8e98826dd1ab4c6d403908dba4bced43%7C5a6d5ee56edf4a26ba93f5872dbb9614%7C0%7C0%7C638284903564485035%7CUnknown%7CTWFpbGZsb3d8eyJWIjoiMC4wLjAwMDAiLCJQIjoiV2luMzIiLCJBTiI6Ik1haWwiLCJXVCI6Mn0%3D%7C3000%7C%7C%7C&sdata=6%2FdZoZ0ofTSVVJclUJX1HQv2XO8UXDhDgO670WpEYc0%3D&reserved=0.
