# Supplementary material for: Dual role of GRHL3 in bladder carcinogenesis depending on histological subtypes
Source: Mol Oncol. 2024 Mar 2;18(6):1397–416. doi: 10.1002/1878-0261.13623 (PMC11164254; doi:10.1002/1878-0261.13623)
Supplement: Supplementary file 1 — Fig. S1. Raw data and uncropped images of western blots presented in Figs 3, 5, and Fig. S3. Fig. S2. Principal component analysis (PCA) of transcriptomic data sets. Fig. S3. GRHL3 overexpression affects colony formation in urothelial J82 cancer cells. Fig. S4. Visualization of enrichment of gene sets involved in integrin complexation in urothelial EJ28 (A, B) and in actin cytoskeleton in SCaBER clones (C, D). Fig. S5. Epithelial cell–matrix adhesion pattern in GRHL3‐expressing SCaBER and EJ28 cells. Fig. S6. Epithelial cell–cell adhesion pattern in GRHL3‐expressing SCaBER and EJ28 cells. Fig. S7. GRHL3 causes downregulation of RHOG in squamous bladder cancer cells. Table S1. Clinicopathological parameters of patients with urinary bladder cancer (n = 264) of the archive of the Institute of Pathology RWTH Aachen analyzed in this study. Table S2. Clinicopathological parameters of patients with non‐muscle‐invasive bladder cancer (NMIBC; n = 107 cases, n = 46 patients) analyzed in this study. Table S3. Primer sequences and PCR conditions. Table S3.1. Primer sequences for RNA analyses. Table S3.2. Mastermix for qPCR. Table S3.3. Cycle conditions of qPCR. Table S3.4. Mastermix for cDNA synthesis. Table S3.5. Cycle conditions of cDNA synthesis. Table S4. Clinicopathological parameters in relation to GRHL3 expression of UC in patient cohort. Table S5. Clinicopathological parameters in relation to GRHL3 expression of sq‐BLCA in patient cohort. Table S6. Clinicopathological parameters in relation to GRHL3 expression of UC in patient cohort. Table S7. Clinicopathological parameters in relation to GRHL3 expression of NMIBC in patient cohort. Table S8. Clinicopathological parameters in relation to GRHL3 expression of UC in patient cohort (NMIBC and MIBC). Table S9. Gene set enrichment analyses of GRHL3‐expressing clones. Table S10. GRHL3 regulated differential expressed gene (DEG) set (adjusted P ≤ 0.05) identified in EJ28 clones. Table S11. GRHL3 regulated differential expresse [file MOL2-18-1397-s001.zip › Supplementary Figures 2-7_R3.docx]

**Supplementary Figures 2-7:**


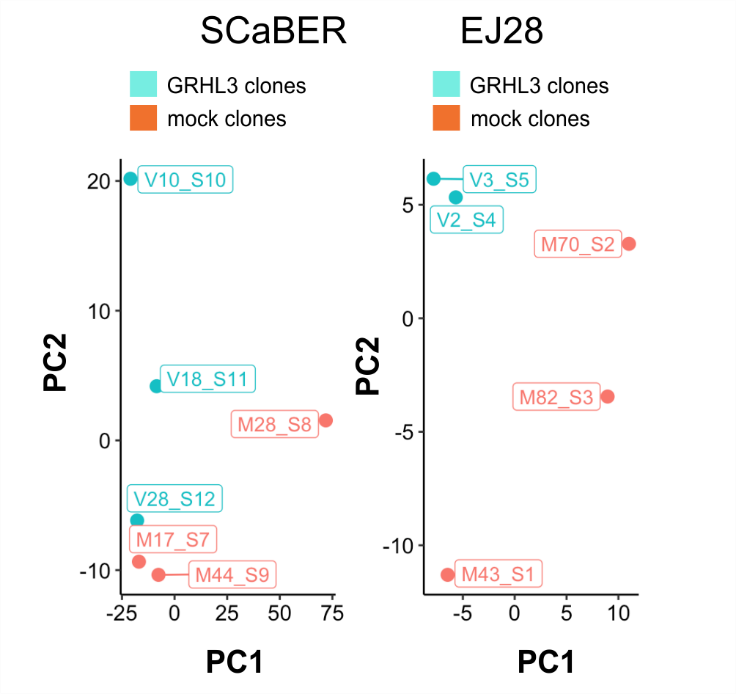


**Supplementary Figure 2. Principal component analysis (PCA) of transcriptomic data sets.** PCA plots were generated by VST (variance stabilizing transformation), normalized for all genes excluding genes with count lower than 5 in all samples which were filtered out.


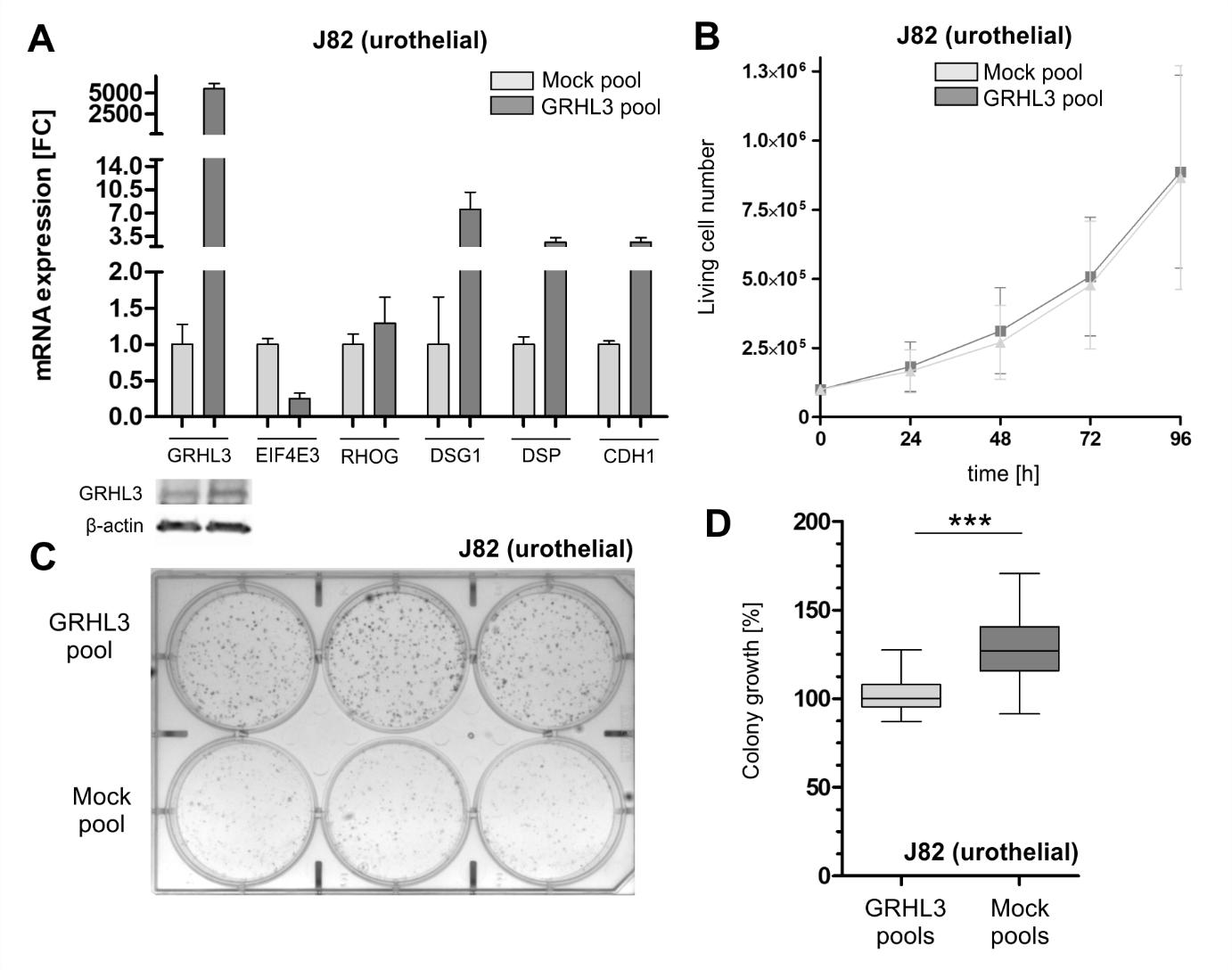


**Supplementary Figure 3. GRHL3 overexpression affects colony formation in urothelial J82 cancer cells.** **(A)** GRHL3 overexpression and putative targets genes in J82 cell line. *Top*: Relative *GRHL3* mRNA expression and target genes (*EIF4E, RHOG, DSG1, DSP, CDH1*) based on real-time PCR comparing mock and stable GRHL3 J82 pools. *Bottom*: Immunoblotting confirms GRHL3 (70 kDa) protein expression in stable GRHL3 J82 pools. β-actin expression served as loading control. **(B)** Short-term cell count assay (n = 4 independent assays) did not show differences in cell proliferation due to GRHL3 expression. **(C-D)** Colony formation assay (n = 5 independent CFAs). (C) Representative colony formation assay of J82 mock and GRHL3 pools. (D) Box plot analyses of colony formation of J82 GRHL3 pools compared to mock pools. ****p < 0.001*. Horizontal lines: grouped medians. Boxes: 25 – 75% quartiles. Vertical lines: range, maximum and minimum.


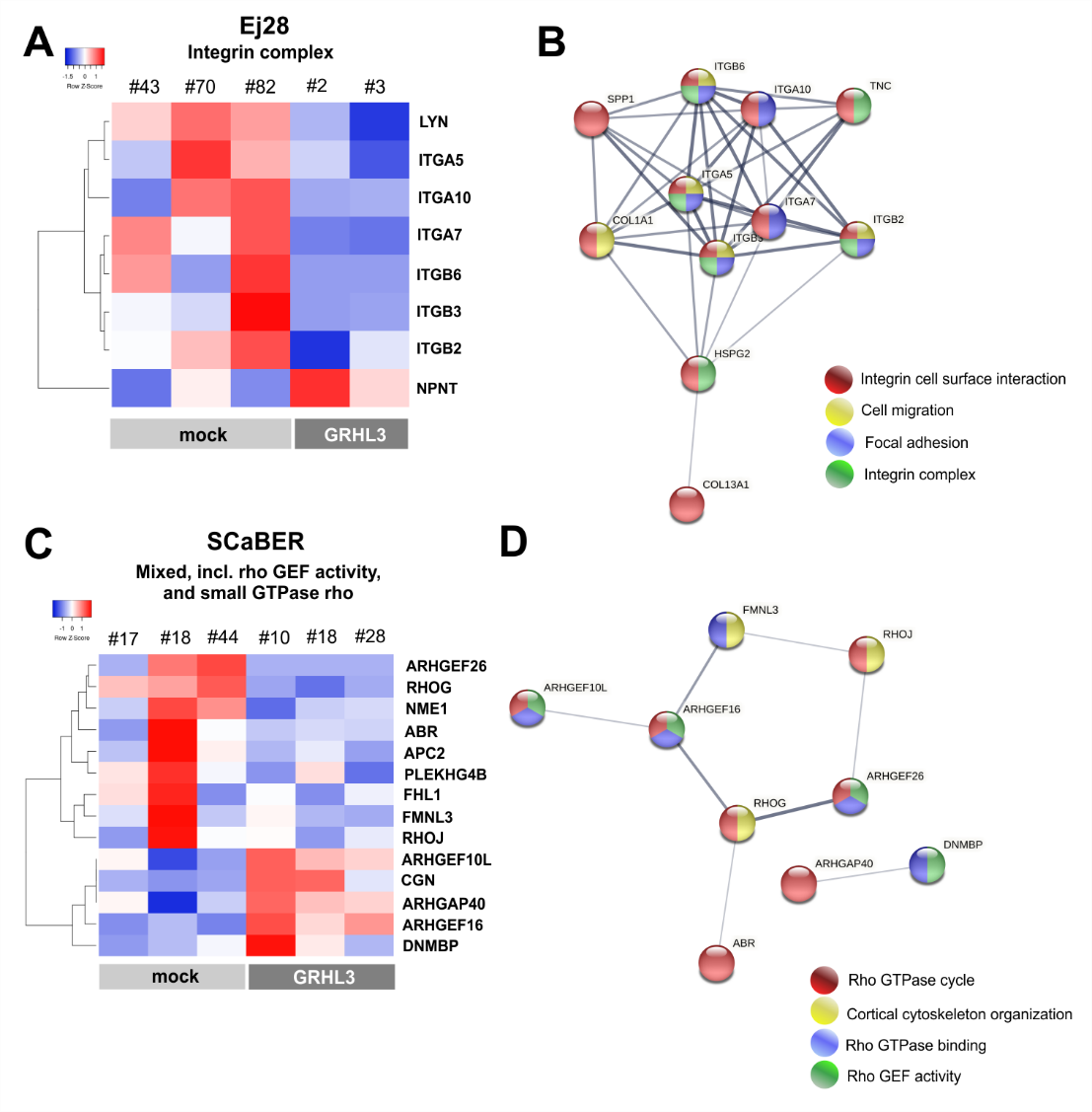


**Supplementary Figure 4. Visualization of enrichment of gene sets involved in integrin complexation in urothelial EJ28 (A-B) and in actin cytoskeleton in SCaBER clones (C-D).** Heatmaps (A, C) highlight mRNA expression of gene sets in analyzed single cell clones of the urothelial and squamous in vitro models. Prediction of putative interaction networks for integrin-mediated cell adhesion (B) and actin cytoskeleton (D) are visualized based on gene sets of heatmaps using the string database.


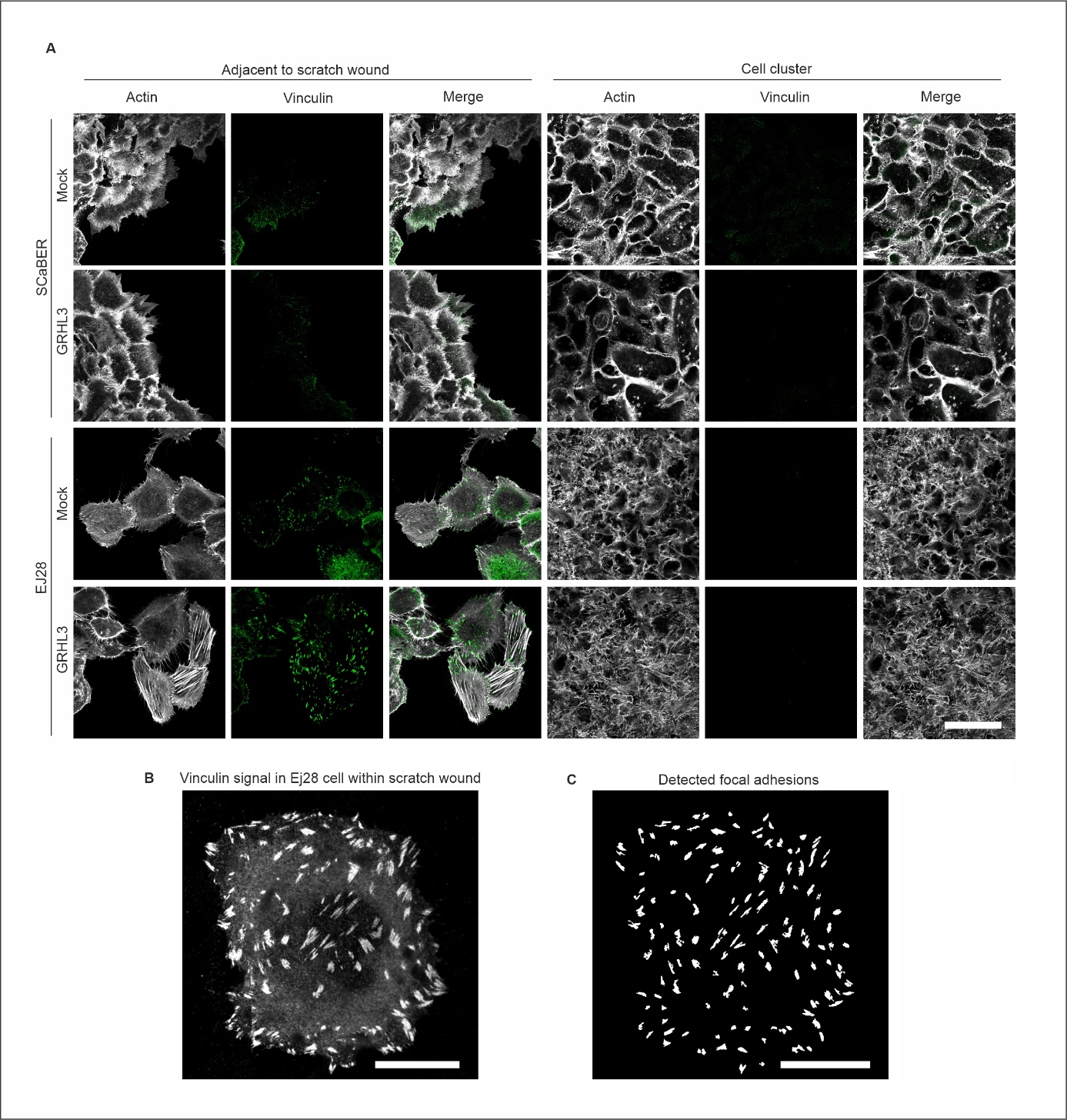


**Supplementary Figure 5:** Epithelial cell-matrix adhesion pattern in GRHL3 expressing SCaBER and EJ28 cells. Cells were fixed and immunostained 16 hours after a scratch wound was applied to confluent cell layers. **(A)** The representative confocal images demonstrate focal adhesion (vinculin, green) formation and co-localization with the actin cytoskeleton (phalloidin, white) at areas of unaffected cell clusters and the edge of the applied scratch Scale bar = 50 µm. **(B)** Micrograph illustrates a single EJ28 cell used to automatically detect and quantify (cf. Figure 5B) focal adhesion number, size and intensity per cell (vinculin, white). **(C)** Corresponsive focal adhesion pattern extracted from the vinculin signal in (B). Scale bars = 20 µm.


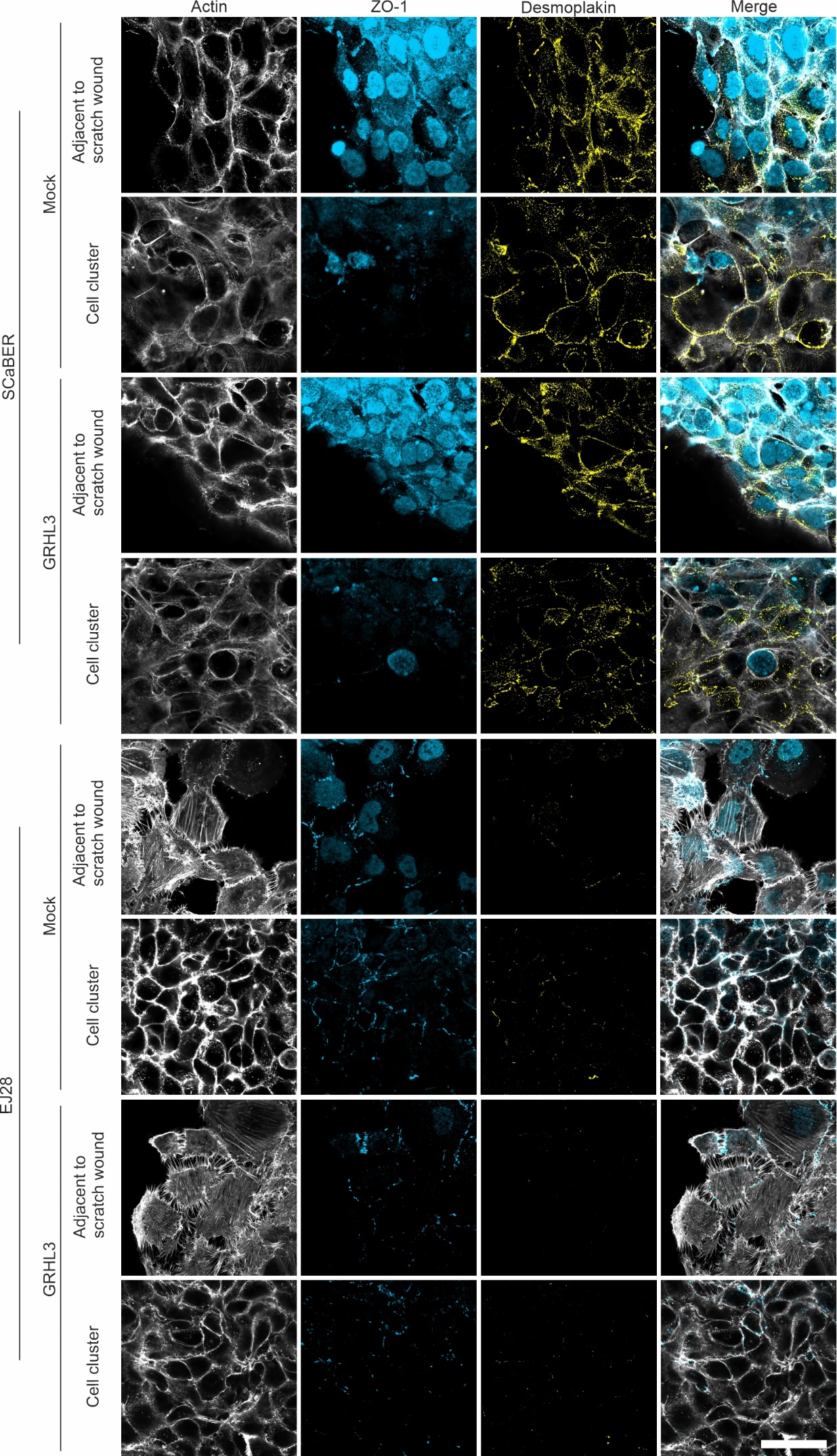


**Supplementary Figure 6:** Epithelial cell-cell adhesion pattern in GRHL3 expressing SCaBER and EJ28 cells. Cells were fixed and immunostained 16 hours after a scratch wound was applied to confluent cell layers. Representative confocal images demonstrate tight junctions (ZO-1, cyan) and desmosomes (desmoplakin, yellow) in conjunction with the actin cytoskeleton (phalloidin, white) at areas of unaffected cell clusters and the edge of the applied scratch. Scale bar = 50 µm.


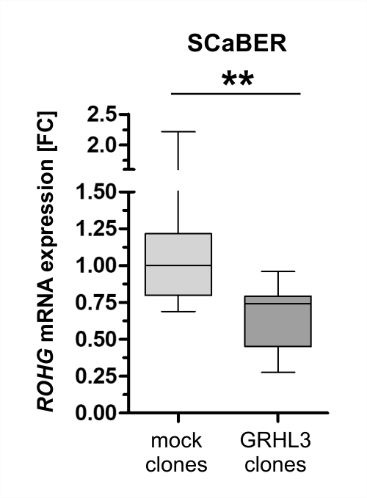


**Supplementary Figure 7:** GRHL3 causes downregulation of RHOG in squamous bladder cancer cells. Inverse regulation between *GRHL3* and *RHOG* mRNA expression in SCaBER single cell clones expressing GRHL3 (each n = 5) compared to control mock clones (each n = 4) using qPCR.
