# Supplementary material for: Dual role of GRHL3 in bladder carcinogenesis depending on histological subtypes
Source: Mol Oncol. 2024 Mar 2;18(6):1397–416. doi: 10.1002/1878-0261.13623 (PMC11164254; doi:10.1002/1878-0261.13623)
Supplement: Supplementary file 1 — Fig. S1. Raw data and uncropped images of western blots presented in Figs 3, 5, and Fig. S3. Fig. S2. Principal component analysis (PCA) of transcriptomic data sets. Fig. S3. GRHL3 overexpression affects colony formation in urothelial J82 cancer cells. Fig. S4. Visualization of enrichment of gene sets involved in integrin complexation in urothelial EJ28 (A, B) and in actin cytoskeleton in SCaBER clones (C, D). Fig. S5. Epithelial cell–matrix adhesion pattern in GRHL3‐expressing SCaBER and EJ28 cells. Fig. S6. Epithelial cell–cell adhesion pattern in GRHL3‐expressing SCaBER and EJ28 cells. Fig. S7. GRHL3 causes downregulation of RHOG in squamous bladder cancer cells. Table S1. Clinicopathological parameters of patients with urinary bladder cancer (n = 264) of the archive of the Institute of Pathology RWTH Aachen analyzed in this study. Table S2. Clinicopathological parameters of patients with non‐muscle‐invasive bladder cancer (NMIBC; n = 107 cases, n = 46 patients) analyzed in this study. Table S3. Primer sequences and PCR conditions. Table S3.1. Primer sequences for RNA analyses. Table S3.2. Mastermix for qPCR. Table S3.3. Cycle conditions of qPCR. Table S3.4. Mastermix for cDNA synthesis. Table S3.5. Cycle conditions of cDNA synthesis. Table S4. Clinicopathological parameters in relation to GRHL3 expression of UC in patient cohort. Table S5. Clinicopathological parameters in relation to GRHL3 expression of sq‐BLCA in patient cohort. Table S6. Clinicopathological parameters in relation to GRHL3 expression of UC in patient cohort. Table S7. Clinicopathological parameters in relation to GRHL3 expression of NMIBC in patient cohort. Table S8. Clinicopathological parameters in relation to GRHL3 expression of UC in patient cohort (NMIBC and MIBC). Table S9. Gene set enrichment analyses of GRHL3‐expressing clones. Table S10. GRHL3 regulated differential expressed gene (DEG) set (adjusted P ≤ 0.05) identified in EJ28 clones. Table S11. GRHL3 regulated differential expresse [file MOL2-18-1397-s001.zip › Supplementary Table 2_FL_NMIBC.docx]

**Supplementary Table 2:** Clinico-pathological parameters of patients with non-muscle-invasive bladder cancer (NMIBC; n = 107 cases, n = 46 patients) analyzed in this study.

| Parameter | Categorization | overall  (n = 107) | | pTa  (n = 88) | | pT1  (n = 19) | |
| --- | --- | --- | --- | --- | --- | --- | --- |
|  |  | **[N]** | **[%]** | **[N]** | **[%]** | **[N]** | **[%]** |
| Gender | Male  Female  unknown | 84  11  12 | 78.51  10.28  11.21 | 71  11  6 | 80.68  12.50  6.82 | 13  0  6 | 68.42  0.00  31.58 |
| Age at diagnosis | Median  Range  50-59  60-69  70-79  80-89  90-99 | 76.00  52.0 – 93.0  3  16  47  15  14 | 2.80  14.95  43.93  14.02  13.08 | 77.00  52.0 – 93.0  2  12  41  14  13 | 2.27  13.63  46.59  15.91  13.00 | 72.00  53.0 – 93.0  1  4  6  1  1 | 5.26  21.05  31.58  5.26  5.26 |
| Tumor grade | G1  G2  G3  G4  unknown | 0  35  72  0  0 | 0.00  32.71  67.29  0.00  0.00 | 0  35  53  0  0 | 0.00  39.77  60.23  0.00  0.00 | 0  0  19  0  0 | 0.00  0.00  100.00  0.00  0.00 |
| Grading | LG  HG | 8  99 | 7.48  92.52 | 8  80 | 9.09  90.91 | 0  19 | 0.00  100.00 |
